# Monitoring Disease Trends using Hospital Traffic Data from High Resolution Satellite Imagery: A Feasibility Study

**DOI:** 10.1038/srep09112

**Published:** 2015-03-13

**Authors:** Elaine O. Nsoesie, Patrick Butler, Naren Ramakrishnan, Sumiko R. Mekaru, John S. Brownstein

**Affiliations:** 1 Children's Hospital Informatics Program, Boston Children's Hospital, Boston, Massachusetts, USA; 2 Department of Pediatrics, Harvard Medical School, Boston, Massachusetts, USA; 3 Department of Epidemiology, Biostatistics and Occupational Health, McGill University, Montreal, Canada; 4 Department of Computer Science, Virginia Tech, Blacksburg, Virginia, USA

## Abstract

Challenges with alternative data sources for disease surveillance include differentiating the signal from the noise, and obtaining information from data constrained settings. For the latter, events such as increases in hospital traffic could serve as early indicators of social disruption resulting from disease. In this study, we evaluate the feasibility of using hospital parking lot traffic data extracted from high-resolution satellite imagery to augment public health disease surveillance in Chile, Argentina and Mexico. We used archived satellite imagery collected from January 2010 to May 2013 and data on the incidence of respiratory virus illnesses from the Pan American Health Organization as a reference. We developed dynamical Elastic Net multivariable linear regression models to estimate the incidence of respiratory virus illnesses using hospital traffic and assessed how to minimize the effects of noise on the models. We noted that predictions based on models fitted using a sample of observations were better. The results were consistent across countries with selected models having reasonably low normalized root-mean-squared errors and high correlations for both the fits and predictions. The observations from this study suggest that if properly procured and combined with other information, this data source could be useful for monitoring disease trends.

Satellite imagery has been used to derive estimates of land use, vegetation index, human and vector population distribution for risk assessment, mapping and forecasting of diseases such as Hantavirus pulmonary syndrome (HPS), malaria, dengue, Lyme, and Rift Valley fever^1^,^2^,^3^,^4^,^5^,^6^,^7^,^8^,^9^,^10^,^11^. These studies have exemplified that if properly analyzed, high-resolution satellite imagery data can be extremely useful for understanding disease spread and implementation of control activities. Remote sensing using satellites has existed as far back as the 1960s and 70s. In contrast, in the last ten to twenty years, numerous studies have advanced several non-traditional data streams as tools to supplement public health surveillance systems. These non-traditional data sources (e.g., social media, micro-blogs, online news reports, and web searches and reservations)^12^,^13^,^14^,^15^,^16^,^17^,^18^,^19^,^20^,^21^,^22^,^23^,^24^ appear to be most suitable for surveillance of diseases with seasonal trends (e.g., influenza, dengue and foodborne diseases) and short incubation periods^20^. However, most surveillance systems based on these data streams depend on the existence of disease reports, mentions of disease-related terms or access to digital disease-related documents. In the case of an emerging infectious disease, the disease signal available through some of these channels might be relatively low due to limitations in public health infrastructure and access to the Internet, thereby limiting (external) real-time monitoring efforts. Other indicators of social disruption such as the number of patients at a hospital with an undiagnosed infection could serve as proxies for early detection of emerging disease outbreaks. Unfortunately, such data are not easily accessible due to bureaucratic, privacy, security and infrastructural reasons.

Data on hospital traffic extracted from satellite imagery of hospital parking lots could serve as an indicator of hospital attendance and could be useful as an estimator of disease activity. In this study, we evaluate the feasibility of using hospital traffic as a possible proxy for detecting influenza and other respiratory illnesses (hereafter referred to as influenza-like illness (ILI)) in Latin American countries. Similar approaches have been used to study and predict hospital admissions due to seasonal diseases^25^, predict hospital occupancy^26^ and to study patterns of hospital use^27^. We estimate hospital traffic based on the number of cars at a hospital parking lot and non-parking lot spaces relative to parking lot size. Data from the Pan American Health Organization (PAHO) is used as a reference for ILI activity. Similar to influenza (and other seasonal respiratory virus) surveillance systems in the United States and several other countries, the release of ILI data to PAHO can be delayed by weeks^28^. The data is also usually updated several weeks after the initial release. This implies that public dissemination of the number of cases due to an emerging outbreak can be delayed by several weeks (or even months) due to delays in reporting, and retrospective updating of case information. The purpose of this study is therefore to present an initial assessment of the use of hospital traffic data in these countries for estimating and predicting disease activity. There are two aims in this study: (1) introduce a new data resource (i.e. high-resolution satellite imagery of hospital traffic) for disease surveillance and (2) evaluate the impact of *recency* (defined as the most recent data observations) in dynamical multivariable linear models for modeling and predicting ILI data from PAHO based on estimates of hospital parking lot occupancy.

## Results

**Figure 1:**
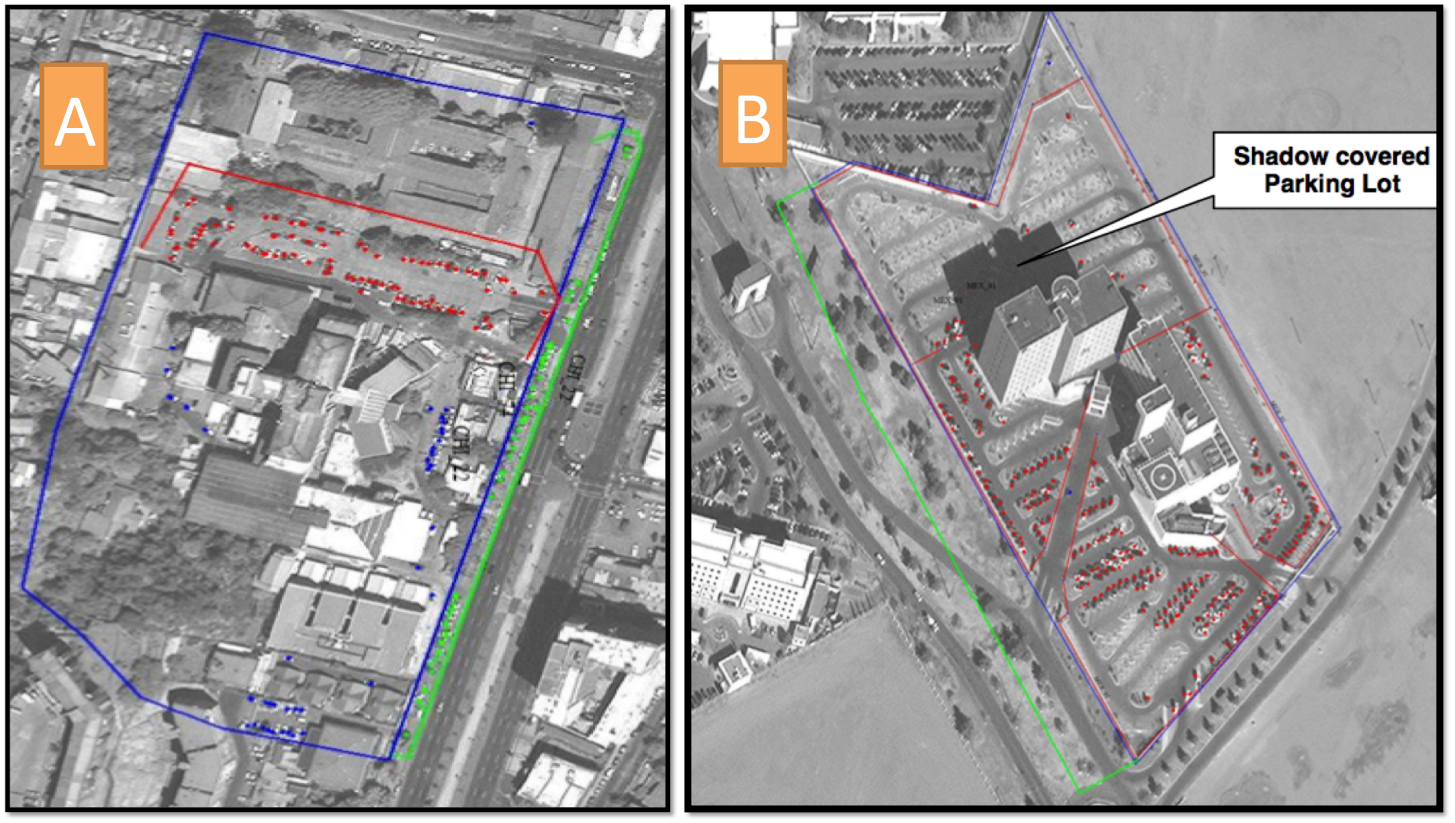
(A) Stencils in different colors were used to delineate hospital premises, parking lot borders and street parking. (B) Example of hospital that was excluded from analysis due to shadow in the parking lot. Remote Sensing Metrics Analysis; Imagery (c) 2014 DigitalGlobe.

After elimination of unsuitable images (example shown in Figure 1), the satellite imagery data consisted of 26, 15 and 13 hospitals for Mexico, Argentina and Chile respectively. We considered four recorded variables (the numbers of vehicles in the parking lot, on the street, and along the hospital border, and the occupancy or fill rate), thereby resulting in 104, 60 and 52 variables respectively. There were 2890 satellite images from January 2010 to May 2013, and all images were used in the analysis. The mean and median numbers of parking lot spaces by country were as follows: Mexico (mean 195, median 155), Argentina (144, 112) and Chile (159, 91).

**Figure 2:**
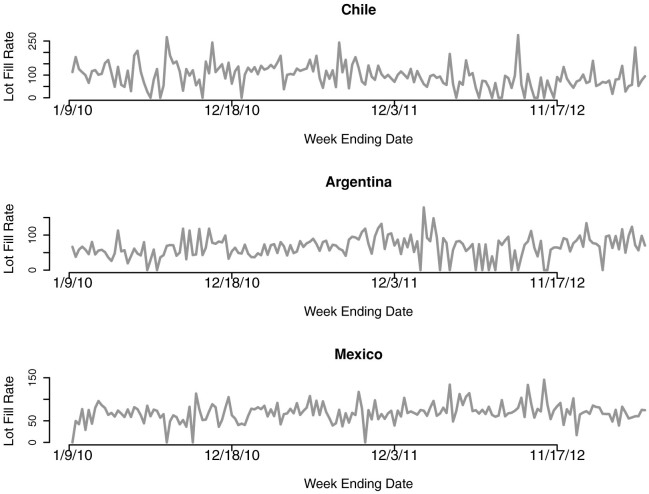
Weekly mean estimates of hospital parking lot fill rate. The fill rate is defined as the number of vehicles in the parking lot, on the street, and along the hospital border divided by the number of available parking spaces.

The mean weekly parking lot occupancy rate is shown in Figure 2. Based on the monthly ILI activity and average number of cars in the parking lot, peaks in parking lot volume appeared to either precede or follow peaks in percent ILI in some cases. For example, ILI activity peaked in the months of September, June and July for 2010, 2012 and 2013 respectively for Chile. In contrast, hospital peak occupancy months were August, March and May respectively. Similarly, for Mexico, hospital peak occupancy was observed in September, May and February, while ILI activity peaked in August, December and January. The trends observed for Argentina were not as consistent. Note that the influenza season typically runs from May to October, and October to May in the Southern and Northern hemispheres respectively. So for most years, for each of the countries, the peak occupancy month fell within the influenza season.

### Recency, Fits and Predictions

**Figure 3:**
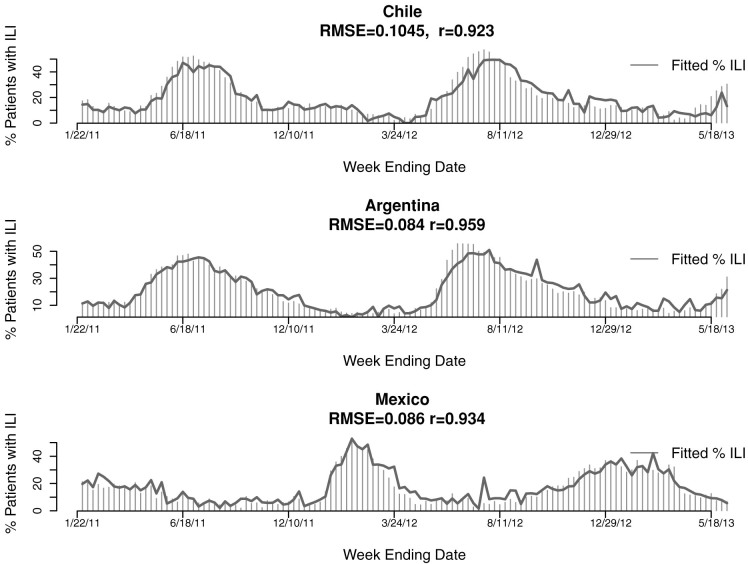
Fit of ILI data to hospital traffic data. At each time point n, all available data from 1 to n were used in model fitting.

**Figure 4:**
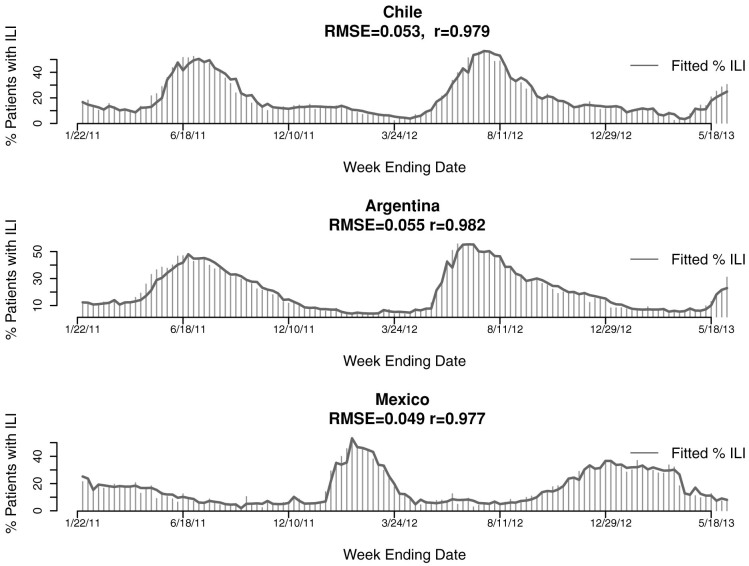
Fit of ILI data to hospital traffic data. At each time point n, the last five data points were used for model fitting (recency = 4). The normalized RMSEs were smaller and Pearson correlation values were higher when compared to Figure 2, for which all observations were used in developing the model.

In Table 1, we present various values of recency (defined as the most recent data observations given by *n − t* to *n*, where *t* is the recency value and n is the current time point) and the resulting normalized root mean squared error (RMSE) and Pearson correlation coefficient between the fitted/predicted values and the percent ILI from PAHO for Chile, Argentina and Mexico. In most cases, the normalized RMSE agreed with the Pearson correlation coefficients. Based on the recency values considered, high correlations between the ILI and fitted/predicted data corresponded to low RMSE values. In addition, smaller recency values appeared to achieve the best fits and predictions based on the correlation and normalized RMSE. The highest correlation and lowest RMSE value pair for the model fits was observed separately at recency values of 4, (4 and 5) and (4 and 5) for Chile, Mexico and Argentina. Note, the model fitted using all the observed data from the initial to the current week (Figure 3) had the lowest correlation and the highest normalized RMSE for the fitted models. The model fitted with a recency value of 4 (Figure 4) had a better fit compared to the model shown in Figure 3. This was consistent across all countries. The fitted models with fewer data points captured the peaks and ILI trend better than the model based on all observations.

**Figure 5:**
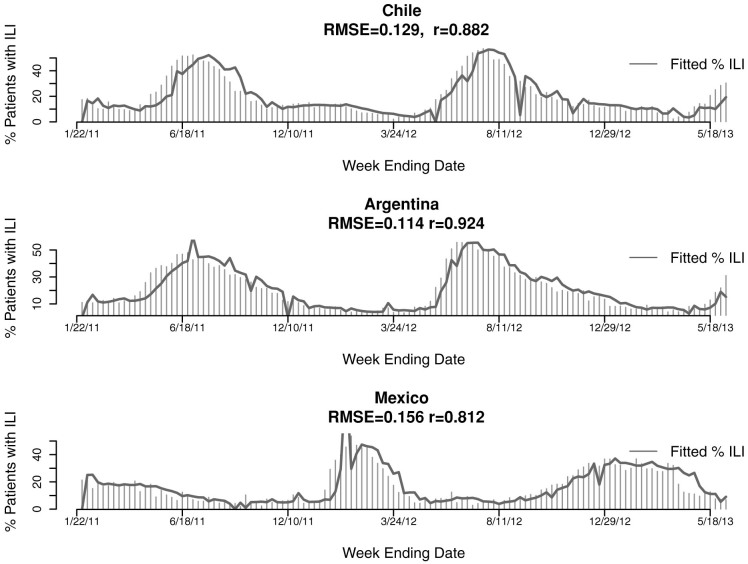
One step-ahead predictions of ILI using hospital traffic data. The lowest normalized root mean squared error and Pearson correlation coefficient pair were observed at different recency values for the different countries.

The correlations and normalized RMSE appeared to depreciate with long-term predictions. For one-step-ahead predictions, the best correlation and RMSE values were individually observed at recency 4 for all countries - Chile, Mexico and Argentina. The best one-step-ahead predictions based on the selected recency values for Chile (RMSE = 0.129; r = 88.2%), Argentina (RMSE = 0.114; r = 92.4%) and Mexico (RMSE = 0.156; r = 81.2%) are presented in Figure 5. The predicted values are lagged especially around the peaks for Chile and Argentina. The model for Mexico over-predicted the peak observed in 2012. Similarly, for two-step-ahead predictions, the best models were observed at recency 4 for Chile, Mexico and Argentina. In general the normalized RMSE and correlations observed were comparable across all countries. Mexico had the most number of hospitals, suggesting there was more data available. However, although Chile had the least number of hospitals, the RMSE and correlations were sometimes better than that for Mexico. This suggests that the performance of the models could partially be explained by the quality of the data and differences in trends across countries rather than the number of observations/images used. Similar observations were made for *recency* values less than 10.

### Hospital Variables

Occupancy for each hospital was represented by the fill rate, number of vehicles in the parking lot, on the street, and along the hospital border. At each week, the elastic net model selected between one and four variables. The number of cars in the parking lot appeared to be the dominant variable (i.e. most significant model coefficient) across all countries. For example, the number of cars in the parking lot of a general care hospital located in the Arica and Parinacota Region had the most significant coefficient for Chile for most weeks when the entire set of observations (Figure 3) and also when the most recent set of observations were used in fitting (as shown in Figure 4). The second most significant coefficient for most weeks was the fill rate of a hospital located in the Metropolitan Region of Chile. The fill rate was also the second most significant variable for the models developed for Mexico and Argentina. Similar to Chile, the hospitals with significant coefficients were located in urban regions specifically, Mexico City for Mexico and Buenos Aires, Ushuaia, and Mendoza for Argentina. The location of the most dominant hospitals in urban areas could be partially explained by the increased likelihood of owning a car in an urban/metropolitan region compared to a rural region.

### Model with Weather Variables

We added weekly mean precipitation, temperature, and absolute humidity as covariates to the models with the highest correlation and smallest RMSE combination. The RMSE and correlation between the fitted values and the PAHO ILI data were (RMSE = 0.048; r = 98.4), (RMSE = 0.043; r = 98.3%), and (RMSE = 0.051; r = 98.5%), for Chile, Mexico and Argentina, respectively. While the RMSE and correlation between the predicted values and the PAHO ILI data were (RMSE = 0.119; r = 89.9%), (RMSE = 0.127; r = 85.6%), and (RMSE = 0.109; r = 93.0), for Chile, Mexico and Argentina, respectively. The fitted and predicted RMSE and correlation are slightly higher when compared to the outcomes from the model solely based on hospital parking lot occupancy data. Absolute humidity was significant at multiple weeks in all three models. The coefficients for precipitation were negative and significant for several weeks in the model for Argentina. In contrast, there were significant negative and positive precipitation coefficients in the models for Chile. Temperature was mildly significant in the Chile model but not significant in the other models.

### Social Unrest

Civil unrest data was available from November 2012 to May 2013. Both negative and positive correlations were observed between reports of civil unrest and hospital traffic, as expected. Civil unrest could affect an increase or decrease in hospital traffic due to injuries or safety concerns. Correlations were in the range (−0.238, 0.235), (−0.360, 0.433) and (−0.482, 0.633) for Chile, Argentina and Mexico respectively. Significant correlations greater than 50% suggest possible associations between trends in hospital traffic and civil unrest events in Mexico.

### Natural Disasters

We focused on Mexico, which had the largest data sample. Using the Welch two sample T-test, we evaluated differences in hospital parking lot occupancy before, during and four weeks after the natural disaster events. The major disasters selected for this analysis were Matthew (tropical storm) in September 23–26, 2010, Fernand (tropical storm) in August 25–26, 2013 and Manuel (category 1 hurricane) in Sept 13–19 2013. These disasters were selected based on the number of individuals affected and reported deaths. For all three situations, there was no statistical significant evidence (*P = 0.391 to 0.9141*) to suggest that hospital parking lot usage was different during and after these disasters.

## Discussion

Our models for influenza and other respiratory viruses using hospital traffic data for select hospitals in Chile, Argentina and Mexico, appear to perform well in capturing the trends present in the data within a reasonable range of error. We used a dynamical Elastic Net approach, which implies that models were fit at each week enabling a dynamical estimation of coefficients and selection of hospital variables that best capture current ILI trends. The models were compared to percent ILI data from PAHO. Ministries of Health and National Influenza Centers of PAHO member states provide the data. The data release is sometimes delayed by a few weeks and data is also retrospectively updated. Therefore, information on current respiratory viruses activity can be delayed by several weeks. Alternative data sources that could serve as early proxies for disease activity are especially useful for monitoring emerging infectious disease outbreaks^29^. For instance, information extracted from satellite images can be processed and available within a few days.

The multivariable models for percent ILI from PAHO based on hospital traffic appear to capture the overall trend and peaks in most instances. However, this seems to depend on the number of recent observations used in developing the models since in most cases, using all observations from the initial to the current week results in spurious peaks and troughs, which leads to higher error rates in both the fits and prediction. One possible reason for these artificial peaks and troughs is that data for each hospital were available at irregular intervals due to the fact that the data was archived and some observations were lost because of factors such as tree cover, building shadow, and construction. Real-time surveillance that involves tasking satellites to take images at particular times of the day would eliminate some of the inconsistency in the data. Images can be taken at multiple times of the day for specific hospitals that best capture disease trends in each country.

In addition, there are expected discrepancies in vehicle ownership when comparing rural versus urban dwellers. So estimating hospital traffic based on the number of cars in the parking lot might not be suitable for rural regions. In addition, parking lots for hospitals in rural areas might be more exposed compared to lots for hospitals in metropolitan regions which might have multiple levels, with only the top level revealed. This could lead to a disproportionate sample of hospitals from urban areas. There are also limitations in the surveillance data used as a reference for ILI activity. Although estimated percent ILI was given for each week, the data available from PAHO has missing values for some viruses. In addition, we also fail to account for other factors that could impact hospital occupancy such as natural disasters (e.g. hurricanes), and social unrest (e.g., riots), and the hospital's distance from a metropolitan region due to lack of data. Although there were some significant correlations between the hospital traffic data and social unrest, defining the duration and scope of impact is challenging. While many projects seek to identify or predict those events through the use of social media or news reports, finding a comprehensive list that can be matched to hospital locations was beyond the scope of this project. Including a flawed list in the model would likely result in extensive misclassification of a binary variable, with the primary concern being false negative values. However, these are variables that ought to be carefully considered in future studies.

Additionally, comparing the fits and predictions, the recency values for the best correlation and RMSE pairs are different. More work is needed to procure satellite data that could best capture the data trends. As with most studies using non-traditional sources of data for disease modeling or predictions, a measure of noise is present. Hence the recency approach might be suitable for developing models in such situations. Recency allows the model to focus on the most recent observations for fitting and predictions. Recent observations of disease incidence are expected to provide the most precise indication of future disease activity/trends. In addition, the most recent observations of the hospital occupancy rates are expected to have the most significant correlation with current disease activity. If satellites are targeted and values recorded more frequently, the sample size would be larger and fewer images would be eliminated during processing.

Other approaches such as syndromic surveillance (e.g., school absenteeism, calls to nurse hotlines, over-the-counter and prescription medication sales) can also be useful for monitoring disease activity in data and resource poor regions^30^. These data sources can supplement limitations in disease surveillance systems by providing early indications of changes in disease and mortality trends. These data sources can also be used in combination with satellite imagery data to improve early detection of disease outbreaks.

The concept of tracking hospital traffic, as an early indicator of disease outbreak especially in the context of limited data availability is promising based on this initial study. However, our study also suggests that if such data sources are to be used as proxies for disease activity, the data procurement needs to be well defined such that the highest quality of data is obtained.

## Methods

### Hospital Traffic Data

We obtained archived high resolution satellite imagery (average resolution of about 70 cm) data of hospital parking lots from Remote Sensing Metrics (RS Metrics), a company that performs quantitative analysis on high-resolution satellite imagery data for various applications^31^. RS Metrics constructed a comprehensive list of hospitals and other healthcare institutions with parking lots for each country (Mexico, Chile and Argentina) using online hospital lists, hospital ranking lists, Google Earth/Google Maps, and Bing Maps. This resulted in a comprehensive list of approximately 120 hospitals and health care facilities for each of the countries (see Supplementary Table 1). Supplementary Table 1 includes information on type of health facility (hospital or other), health care provider (private or government), location (rural or urban), number of beds (if available) and hospital ranking (if available). Upon initial analysis (not presented), we limited the hospital list to: (i) non-specialty (or general care) hospitals and eliminated specialty hospitals (such as psychiatric hospitals, and surgical clinics) and research centers based on information provided on each hospital (or health entity) website; (ii) hospitals with more than forty parking spaces to increase the chance of detecting significant anomalies in hospital traffic.

For each hospital, RS Metrics performed automated data extraction by first delineating hospital premises, parking lot borders and street parking in different colors as shown in Figure 1A. Images with tree cover, building shadow (e.g., Figure 1B), construction and other factors that present difficulties in defining the contours were excluded since this could lead to over- or under-counting of the number of vehicles. After delineation, the company used a standard approach for processing images for all clients. This involved a combination of Automated Feature Extraction (AFE) software, manual counting and quality control, and workflow management software to count the number of cars and parking spaces. Please note that the process of data analysis was independent of the image selection process.

The dataset used in analysis consisted of the date and time of each image; the hospital's name and geographic location (including the address, latitude, and latitude); the numbers of vehicles in the parking lot, on the street, and along the hospital border; the number of parking lot spaces and the occupancy or fill rate defined as the number of cars divided by the number of available parking spaces. We obtained weekly estimates for each variable by averaging over weeks with multiple observations and used data from January 2010 to May 2013 in our analysis.

### PAHO Data

PAHO compiles data on weekly levels of ILI activity for member states based on data submitted by the Ministries of Health (MOH) and National Influenza Centers (NCI), or updates extracted from MOH webpages of member states. The data is openly available via the PAHO-WHO Influenza and other Respiratory Viruses Surveillance tool: http://ais.paho.org/phip/viz/ed_flu.asp and downloadable at a weekly resolution. As of Thursday September 4^th^, 2014, the list of viruses included in the ILI data consisted of Influenza A (H3N2), Flu A (H1N1) pdm09, Flu A Not Subtyped, Flu A Not Subtypeable, Influenza B, Adenovirus, Parainfluenza, Respiratory Syncytial Virus (RSV), Bocavirus, Coronavirus, Metapneumovirus, Rhinovirus and other viruses (not listed). We downloaded weekly data for Argentina, Mexico and Chile for the same time period as the satellite imagery data: January 2010 to May 2013.

### Weather Data

In addition to disease, weather, social unrest and natural disasters are other factors that could influence hospital traffic. We obtained temperature, absolute humidity and precipitation data from the Global Data Assimilation System (GDAS). The data was extracted in GRIB format from http://ladsweb.nascom.nasa.gov/ at a one-degree latitude/longitude resolution for each of the countries – Chile, Mexico and Argentina. These weather covariates were selected because they can influence decisions on car usage and studies have shown associations between absolute humidity and onset of influenza epidemics^32^,^33^. Each of the meteorological covariates was averaged at a weekly level and time-series were constructed from January 2010 to May 2013.

### Civil Unrest and Natural Disasters

The civil unrest data was extracted from openly available data sources (e.g., government reports, social media (such as Twitter) and newspaper reports). The dataset had been used by Doyle et al.^34^ in a project aimed at producing real-time detailed forecasts of future events. The civil unrest events included planned protests and riots. Due to the scope of project reported in Doyle et al.^34^ the data was limited to November 2012 to May 2013. We used Pearson correlation to evaluate any associations between frequency of civil unrest reports and trends in hospital traffic.

Natural disasters may include earthquakes, hurricanes, floods and fires. Although the exact time and location of an earthquake or hurricane landfall may be precisely determined, definition of the duration and scope of impact is more challenging. To evaluate potential associations between natural disasters and the hospital traffic data, we selected three major natural disasters for Mexico and assessed differences in mean hospital parking lot occupancy four weeks before, during and immediately following the event using the Two Sample Welch T-test. We focused on Mexico since it had the largest data sample.

### Multivariable Regression Model

We developed multivariable linear regression models to estimate and predict weekly percent ILI for Mexico, Chile and Argentina.
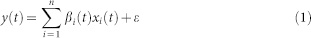
Hospital occupancy reflected by each of the variables (fill rate, number of vehicles in the parking lot, on the street, and along the hospital border) for each hospital is represented as a single explanatory variable *x_i_*. PAHO percent of hospital/clinic visits with ILI (hereafter referred to as percent ILI) is the dependent variable *y*, *α_i_* are the coefficients and the normally distributed error term is given by *ε*. The number of variables *n* varies since the number of hospitals varies by country.

We used the Elastic Net regularization and variable selection method^35^ to select the hospital variables that best captured the trend in the ILI data. The elastic net estimator is given by:
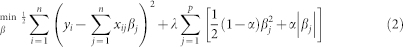
The elastic net combines the properties of the Least Absolute Shrinkage and Selection Operator (LASSO) and Ridge regression procedures. When *α* equals to 0 and 1, (2) equates to the Ridge and LASSO estimators respectively. The LASSO procedure minimizes the sum of squared errors subject to a bound on the sum of the absolute values of the coefficients^36^. Ridge regression has a grouping effect, whereby it tends to select all correlated variables. The elastic net combines these two properties such that it tends to select and average the coefficients of highly correlated predictors if any of the variables within the group is selected. The procedure performs well for studies were the number of covariates is greater than the number of observations (p ≫ n). In such a situation, the number of selected variables can be greater than the number of observations. We make use of this property by fitting models to different sample sizes as later discussed.

Correlations between hospital variables differed by country, which could require different values for *α*. Models for all three countries were fit with *α* at 0.8 after exploring values between 0.5 and 0.9. At each data observation (i.e., each week), each of the model coefficients are updated so as to continuously select a subset of variables that provides the best model fit. This results in a diversity of hospital variables used in the model at each week. The model selected by elastic net for each week was used in one and two step-ahead predictions of the weekly percent ILI.

Since the data was extracted from a historical archive and not based on targeting satellites to specific locations, and due to the elimination of images deemed unsuitable, the data had some missing observations. These missing observations were filled using the last known value. To improve the prediction and reduce the impact of noise in our models, we fitted models using a range of previous values (henceforth referred to as *recency*). We defined recency as the most prior weeks of data given by *n − t* to *n*, where *t* is the recency value and n is the current week. This can be illustrated as follows. Let recency equals to *t* and *S* represent the complete training set:
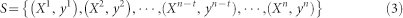
Then the recency sample is defined as:
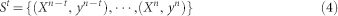
We considered a range of small and large recency values. Given that we expect recent changes in parking lot usage to correlate with recent changes in disease activity, we selected values that were between 4, 5, 6, and 7 weeks so the number of observations for each covariate was at least five. We later assessed whether similar observations could be made if the analysis focused on the last three, six and twelve months of data. For consistency, across all recency values, the initial model was fitted starting from the third week in 2011 and the model fits and predictions were compared based on the normalized root mean squared error (RMSE) and the Pearson correlation coefficient (r). The first set of models solely used hospital parking lot occupancy variables as covariates. The second set of models considered both the hospital parking lot data and meteorological covariates. Model parameters were estimated using a ten-fold cross validation approach and the models were implemented using the glmnet package in the R statistical software.

## Author Contributions

E.O.N. and P.B. analyzed the data. E.O.N, P.B., N.R., S.R.M., and J.S.B. wrote the manuscript. All authors reviewed the manuscript

## Supplementary Material

Supplementary Information Supplementary tables

## Figures and Tables

**Table 1:** Models fit and predictions at different recency values. The outcomes were compared based on the Pearson correlation coefficient represented by r, and the normalized root mean squared error, given as RMSE. Note, for all countries, the model fitted using all observations had the smallest r and highest RMSE

		Fitting		Prediction: 1 Step ahead		Prediction: 2 Step ahead	
Country	Recency (weeks)	r	RMSE	r	RMSE	r	RMSE
Chile	4	0.979	0.053	0.882	0.129	0.800	0.169
	5	0.977	0.058	0.842	0.149	0.724	0.198
	6	0.975	0.060	0.829	0.158	0.618	0.243
	7	0.968	0.067	0.740	0.196	0.570	0.259
	13	0.949	0.084	0.672	0.221	0.548	0.269
	26	0.962	0.079	0.750	0.185	0.549	0.251
	52	0.967	0.071	0.864	0.136	0.757	0.179
	None	0.923	0.1045	0.744	0.190	0.579	0.247
Argentina	4	0.982	0.055	0.924	0.114	0.840	0.164
	5	0.984	0.053	0.880	0.142	0.812	0.178
	6	0.980	0.058	0.913	0.120	0.828	0.171
	7	0.981	0.057	0.897	0.132	0.810	0.178
	13	0.964	0.078	0.850	0.156	0.704	0.222
	26	0.970	0.073	0.826	0.168	0.630	0.261
	52	0.976	0.068	0.810	0.173	0.665	0.229
	None	0.959	0.084	0.821	0.169	0.710	0.218
Mexico	4	0.977	0.049	0.812	0.156	0.763	0.176
	5	0.968	0.059	0.800	0.156	0.729	0.189
	6	0.975	0.052	0.770	0.173	0.727	0.186
	7	0.965	0.061	0.765	0.173	0.598	0.218
	13	0.975	0.053	0.725	0.166	0.568	0.222
	26	0.975	0.053	0.691	0.180	0.430	0.248
	52	0.960	0.067	0.687	0.181	0.480	0.240
	None	0.934	0.086	0.495	0.251	0.341	0.311
