# Supplementary material for: Monitoring Disease Trends using Hospital Traffic Data from High Resolution Satellite Imagery: A Feasibility Study
Source: Sci Rep. 2015 Mar 13;5:9112. doi: 10.1038/srep09112 (PMC4357853; doi:10.1038/srep09112)
Supplement: Supplementary Information — Supplementary tables [file srep09112-s1.pdf]

Monitoring Disease Trends using Hospital Traffic Data from High Resolution Satellite Imagery: A Feasibility Study

Elaine O. Nsoesie[1,2], Patrick Butler[4], Naren Ramakrishnan[4] , John S. Brownstein[1,2,3]

1. Children's Hospital Informatics Program, Boston Children's Hospital, Boston, Massachusetts, USA
2. Department of Pediatrics, Harvard Medical School, Boston, Massachusetts, USA
3. Department of Epidemiology, Biostatistics and Occupational Health, McGill University, Montreal, Canada
4. Department of Computer Science, Virginia Tech, Blacksburg, Virginia, USA

Table 1. List of healthcare facilities generated by RS Metrics for each country in the study.

Hospital Full Name	Hospital Address	Hospital or Not	Public or Private?	Rural or Urban?	Number of Beds	Ranking	Website
Hospital General De Agudos Juan Fernandez	Avenida Cerviño 3356, C1425AGP Buenos Aires, Argentina	Hospital - includes Cardiology, Surgery, Urgent Care, Clinical Medicine and Infant and OB-GYN departments	Public	Urban	**442** (2014)	26	http://www.fundacionfernandez.org/el_hospital.html
Hospital General de Agudos De Bernardino Rivadavia	Avenida Gral. Las Heras 2670, C1425ASQ Buenos Aires, Capital Federal, Argentina	Hospital	Public	Urban	**306** (2001)		http://www.buenosaires.gob.ar/areas/salud/rivadavia/
Hospital Materno Infantil Ramon Sarda	Esteban de Luca 2151, C1246ABQ Buenos Aires, Capital Federal, Argentina	Not - only pediatric, family and "generalist" doctors focused on perinatology	Public	Urban	**100** (2014? http://www.sarda.org.ar/Home/Resena_Historica)	3	http://www.sarda.org.ar/Home/Resena_Historica
	Gral. José María Paz Este 998, San Juan, San Juan Province, Argentina	Hospital - includes Anesthesiology, General Surgery, OB-GYN, Dialisis, Pediatrics, Neuro, among other departments	Public	Urban	**520** (no date http://www.laseptima.info/noticias/15493)		No website
	Leandro N. Alem 1450, Rosario, Santa Fe Province, Argentina	General Hospital	Public (Official Provincial)	Urban	?		No website
	Avenida Belgrano 2975, C1209AAB Buenos Aires, Argentina		Private				http://www.hospital-espanol.com.ar/
	Urquiza 3100, 2000 Rosario, Santa Fe Province, Argentina	Appears to be a Laboratory/Research center funded by a Foundation					http://www.rosario.gov.ar/sitio/paginainicial/

Hospital General de Ninos Ricardo Gutierrez	Sánchez de Bustamante 1330, C1425EFD Buenos Aires, Capital Federal, Argentina	Hospital - provides toxicology for adults and pediatrics including pediatric cardiology, dermatology, neurology, gastroenterology, psychiatry, etc (http://www.buenosaires.gob.ar/areas/salud/sistemas_salud/ficha.php?id=34)			?	22	No website
Hospital Dr. Ramon Carrillo -Centro de Asistencia y Rehab in Salud Mental en Posadas	Dr. Ramón Carrillo 375, C1275AHG Buenos Aires, Capital Federal, Argentina	Hospital for Mental Health	Public	Urban	?		No Website
	Avenida Luis María Campos 726, Buenos Aires, Buenos Aires Province, Argentina	Military Hospital	Military personnel and their families	Urban	?		http://www.hmc.mil.ar/
	Pellegrini Avenue 3205, Rosario, Santa Fe Province, Argentina	Hospital and Emergency/Trauma Center	Public	Urban	**155** (according to Wikipedia http://es.wikipedia.org/wiki/Hospital_de_Emergencias_Dr._Clemente_%C3%81lvarez)		http://www.fundacionheca.org.ar/hospital/index.php?option=com_content&view=article&id=1&Itemid=2
	Perdriel 74, C1280AEB Buenos Aires, Argentina	Hospital	Private (?)	Urban	?	10	http://www.hospitalbritanico.org.ar/web/es/el-hospital/hospital-central-1
	Córdoba 4545, Mar del Plata, Buenos Aires Province, Argentina	Hospital	Private (?)	Urban	**240** (https://www.youtube.com/watch?v=FCjeg9OepDk)	5	http://www.hpc.org.ar/inicio
Hospital Naval Pedro Mallo	Avenida Patricias Argentinas, Buenos Aires, Buenos Aires Province, Argentina	Unclear whether or not it is a functioning hospital	Was for Navy Personel and their Families				http://www.hnpm.mil.ar/
Hospital Zonal Dr. Ramon Carrillo or Hospital Bariloche	San Carlos de Bariloche, Río Negro Province, Argentina	Hospital - General Medicine, General Surgery, Tocogynecology, Clinical Medicine, Pediatrics and Emergency	Public	Urban	**146** (http://www.hospitalbariloche.com.ar/datos.htm)		http://www.hospitalbariloche.com.ar/
Hospital Santojanni	Pilar 950, 1408 Capital Federal, City of Buenos Aires, Buenos Aires Province, Argentina	Hospital with 7 Community Health Centers	Public	Urban	?		http://www.hospitalsantojanni.gov.ar/web/index.php/elhospital/2011-08-15-05-52-35/historia

Hospital General de Agudos Teodoro Alvarez	Dr. Juan Felipe Aranguren 2701, C1406FWY Buenos Aires, Capital Federal, Argentina	Hospital - Surgery, Emergency, Medicine, Pediatrician, OB/GYN Departments	Public	Urban	336		http://www.hospitalalvarez.org/index.php
	Diagonal 75, La Plata, Buenos Aires Province, Argentina	Children's Hospital	Public	Appears Suburban	350	21	http://www.ludovica.org.ar/inicio.html
	Buenos Aires, Argentina	Mental Health Hospital	?	Urban	?		http://www.buenosaires.gob.ar/hospitaltobargarcia
Hospital Zonal Gral Agudos Dr Isidoro Iriate	Quilmes, Buenos Aires Province, Argentina	Hospital	Public	Urban	221		None but I found this: http://www.argentino.com.ar/hospital-zonal-general-agudos-dr-isidoro-iriarte-F1506C80F18D2
Casa Hospital San Juan de Dios - Ramos Mejia	Ramos Mejía, Buenos Aires Province, Argentina	Hospital	Private	Urban	**78** (http://www.hsjd.org/arg-ramos/centros-arg-ramos.php)		http://www.hsjd.org/hospital/index.php
Hospital Area Programa Choele Choel	Choele Choel, Río Negro Province, Argentina	Hospital	?	?	**70** (http://www.argentino.com.ar/hospital-area-programa-choele-choel-F140FC80713D4)		no website
	Calle 9, 1900 La Plata, Buenos Aires Province, Argentina		Private				
Hospital Espanol de Mendoza	Avenida San Martín 965, Godoy Cruz, Mendoza Province, Argentina	Hospital	Private	Ubran	**200** (http://www.hespanol.com.ar/paginas/index/infraestructura)	37	http://www.hespanol.com.ar/paginas/index/mision-vision-y-objetivos
Hospital General de Agudos Carlos Durand	Avenida Díaz Vélez 5044, C1405DCS Buenos Aires, Argentina	Hospital	Public	Urban	**326** (2001 http://www.wilsoncenter.org/sites/default/files/INFORME_HOSPITALES_DE_AGUDO.PDF)		http://www.hospitaldurand.org.ar/
	San Juan 2021, C1232AAC Buenos Aires, Capital Federal, Argentina						
Hospital General de Agudos Dr Cosme Argerich	Pi y Margall 750, C1155AHD Buenos Aires, Argentina	Hospital	Public	Urban	**400** (http://www.hospitalargerich.org.ar/)		http://www.hospitalargerich.org.ar/

Hospital Privado Centro Medico de Cordoba	Naciones Unidas 346, Córdoba, Córdoba Province, Argentina	Hospital	? (I would guess private)	Urban	**268** (http://www.hospitalprivadosa.com.ar/hospital/infraestructura.htm)	12	http://www.hospitalprivadosa.com.ar/
	Río Grande, Argentina						
Hospital Italiano de Cordoba	Roma 550, Córdoba, Córdoba Province, Argentina	Hospital	Private	Urban	**240** (http://www.hospital-italiano.com.ar/hospital-italiano/institucional/info)	31	http://www.hospital-italiano.com.ar/hospital-italiano/
Casa Hospital San Juan de Dios - Ramos Mejia	Ramos Mejía, Buenos Aires Province, Argentina	Hospital	Private	Urban	**78** (http://www.hsjd.org/arg-ramos/centros-arg-ramos.php)		http://www.hsjd.org/hospital/index.php
Hospital Area Programa Choele Choel	Choele Choel, Río Negro Province, Argentina	Hospital	?	?	**70** (http://www.argentino.com.ar/hospital-area-programa-choele-choel-F140FC80713D4)		no website
	Calle 9, 1900 La Plata, Buenos Aires Province, Argentina		Private				
Hospital Espanol de Mendoza	Avenida San Martín 965, Godoy Cruz, Mendoza Province, Argentina	Hospital	Private	Urban	**200** (http://www.hespanol.com.ar/paginas/index/infraestructura)	37	http://www.hespanol.com.ar/paginas/index/mision-vision-y-objetivos
Hospital General de Agudos Carlos Durand	Avenida Díaz Vélez 5044, C1405DCS Buenos Aires, Argentina	Hospital	Public	Urban	**326** (2001 http://www.wilsoncenter.org/sites/default/files/INFORME_HOSPITALES_DE_AGUDO.PDF)		http://www.hospitaldurand.org.ar/
	San Juan 2021, C1232AAC Buenos Aires, Capital Federal, Argentina						
Hospital General de Agudos Dr Cosme Argerich	Pi y Margall 750, C1155AHD Buenos Aires, Argentina	Hospital	Public	Urban	**400** (http://www.hospitalargerich.org.ar/)		http://www.hospitalargerich.org.ar/
Hospital Privado Centro Medico de Cordoba	Naciones Unidas 346, Córdoba, Córdoba Province, Argentina	Hospital	? (I would guess private)	Urban	**268** (http://www.hospitalprivadosa.com.ar/hospital/infraestructura.htm)	12	http://www.hospitalprivadosa.com.ar/

	Río Grande, Argentina						
Hospital Italiano de Cordoba	Roma 550, Córdoba, Córdoba Province, Argentina	Hospital	Private	Urban	**240** (http://www.hospital-italiano.com.ar/hospital-italiano/institucional/info)	31	http://www.hospital-italiano.com.ar/hospital-italiano/
	Perdriel 74, C1280AEB Buenos Aires, Argentina	Hospital	Private (?)	Urban	?	10	http://www.hospitalbritanico.org.ar/web/es/el-hospital/hospital-central-1
	Pte. Illia s/n, 1684 El Palomar, Buenos Aires, Argentina	Hospital				11	http://www.hospitalposadas.gov.ar/index.php
	Juan D. Peron 4190, C1181ACH Buenos Aires, Argentina	2 Hospital Facilities	Private	Urban	**750** beds (http://www.imia-medinfo.org/new2/node/287)	1	http://www.hospitalitaliano.org.ar/
	Avenida Pueyrredón 1640, C1118AAT Buenos Aires, Capital Federal, Argentina	Hospital	Private	Urban	**254** (http://es.wikipedia.org/wiki/Hospital_Alem%C3%A1n)	16	http://www.hospitalaleman.org.ar/
	Luis Monti 155, Zapala, Neuquén Province, Argentina	Hospital	?	?	?		http://hospitalzapala.org/
Hospital Pedro de Elizalde	Avenida Manuel Montes de Oca 40, C1270AAN Buenos Aires, Capital Federal, Argentina	Pediatric Hospital	Public	Urban	**290** (http://www.hospitalelizalde.org/area_medica/plantel/serv_dire.asp)	27	http://www.hospitalelizalde.org/hosp.asp
Hospital de Clinicas de Jose De San Martin	2351, Buenos Aires, Buenos Aires Province, Argentina	Hospital	Public	Urban	**400** (http://es.wikipedia.org/wiki/Hospital_de_Cl%C3%ADnicas_Jos%C3%A9_de_San_Mart%C3%ADn)	34	http://www.hospitaldeclinicas.uba.ar/
Hospital General de Agudos Pirovano	Avenida Monroe 3555, C1430BKC Buenos Aires, Capital Federal, Argentina	Hospital	Public	Urban	**382** (2001: http://www.wilsoncenter.org/sites/default/files/INFORME_HOSPITALES_DE_AGUDO.PDF)		http://www.buenosaires.gob.ar/areas/salud/sistemas_salud/ficha.php?id=29
	Avenida Juan Domingo Perón 1500, 1629, Buenos Aires Province, Argentina	Hospital	University Private	Rural	**136** (2014: http://rankings.americaeconomia.com/mejores-clinicas-hospitales-2014/ranking/)	14	http://www.hospitalaustral.edu.ar/home.asp

Hospital General de Agudos Ramos Mejia	General Urquiza 609, C1221ADC Buenos Aires, Capital Federal, Argentina	Hospital	Public	Urban	**504** (2001: http://www.wilsoncenter.org/sites/default/files/INFORME_HOSPITALES_DE_AGUDO.PDF)		http://www.hospitalramosmejia.info/
Hospital General De Agudos Juan Fernandez	Avenida Cerviño 3356, C1425AGP Buenos Aires, Argentina	Hospital - includes Cardiology, Surgery, Urgent Care, Clinical Medicine and Infant and OB-GYN departments	Public	Urban	**442** (2014)	26	http://www.fundacionfernandez.org/el_hospital.html
Hospital General de Agudos De Bernardino Rivadavia	Avenida Gral. Las Heras 2670, C1425ASQ Buenos Aires, Capital Federal, Argentina	Hospital	Public	Urban	**306** (2001)		http://www.buenosaires.gob.ar/areas/salud/rivadavia/
Hospital Materno Infantil Ramon Sarda	Esteban de Luca 2151, C1246ABQ Buenos Aires, Capital Federal, Argentina	Not - only pediatric, family and "generalist" doctors focused on perinatology	Public	Urban	**100** (2014? http://www.sarda.org.ar/Home/Resena_Historica)		http://www.sarda.org.ar/Home/Resena_Historica
	Gral. José María Paz Este 998, San Juan, San Juan Province, Argentina	Hospital - includes Anesthesiology, General Surgery, OB-GYN, Dialisis, Pediatrics, Neuro, among other departments	Public	Urban	**520** (no date http://www.laseptima.info/noticias/15493)		No website
	Leandro N. Alem 1450, Rosario, Santa Fe Province, Argentina	General Hospital	Public (Official Provincial)	Urban	?		No website
	Avenida Belgrano 2975, C1209AAB Buenos Aires, Argentina		Private				http://www.hospital-espanol.com.ar/
	Urquiza 3100, 2000 Rosario, Santa Fe Province, Argentina	Appears to be a Laboratory/Research center funded by a Foundation					http://www.rosario.gov.ar/sitio/paginainicial/
Hospital General de Ninos Ricardo Gutierrez	Sánchez de Bustamante 1330, C1425EFD Buenos Aires, Capital Federal, Argentina	Hospital - provides toxicology for adults and pediatrics including pediatric cardiology, dermatology, neurology, gastroenterology, psychiatry, etc (http://www.buenosaires.gob.ar/areas/salud/sistemas_salud/ficha.php?id=34)			?		No website

Hospital Dr. Ramon Carrillo -Centro de Asistencia y Rehab in Salud Mental en Posadas	Dr. Ramón Carrillo 375, C1275AHG Buenos Aires, Capital Federal, Argentina	Hospital for Mental Health	Public	Urban	?		No Website
	Avenida Luis María Campos 726, Buenos Aires, Buenos Aires Province, Argentina	Military Hospital	Military personnel and their families	Urban	?		http://www.hmc.mil.ar/
	Pellegrini Avenue 3205, Rosario, Santa Fe Province, Argentina	Hospital and Emergency/Trauma Center	Public	Urban	**155** (according to Wikipedia http://es.wikipedia.org/wiki/Hospital_de_Emergencias_Dr._Clemente_%C3%81lvarez)		http://www.fundacionheca.org.ar/hospital/index.php?option=com_content&view=article&id=1&Itemid=2
	Perdriel 74, C1280AEB Buenos Aires, Argentina	Hospital	Private (?)	Urban	?	10	http://www.hospitalbritanico.org.ar/web/es/el-hospital/hospital-central-1
	Córdoba 4545, Mar del Plata, Buenos Aires Province, Argentina	Hospital	Private (?)	Urban	**240** (https://www.youtube.com/watch?v=FCjeg9OepDk)	5	http://www.hpc.org.ar/inicio
Hospital Naval Pedro Mallo	Avenida Patricias Argentinas, Buenos Aires, Buenos Aires Province, Argentina	Unclear whether or not it is a functioning hospital	Was for Navy Personel and their Families				http://www.hnpm.mil.ar/
Hospital Zonal Dr. Ramon Carrillo or Hospital Bariloche	San Carlos de Bariloche, Río Negro Province, Argentina	Hospital - General Medicine, General Surgery, Tocogynecology, Clinical Medicine, Pediatrics and Emergency	Public	Urban	**146** (http://www.hospitalbariloche.com.ar/datos.htm)		http://www.hospitalbariloche.com.ar/
Hospital Santojanni	Pilar 950, 1408 Capital Federal, City of Buenos Aires, Buenos Aires Province, Argentina	Hospital with 7 Community Health Centers	Public	Urban	?		http://www.hospitalsantojanni.gov.ar/web/index.php/elhospital/2011-08-15-05-52-35/historia
Hospital General de Agudos Teodoro Alvarez	Dr. Juan Felipe Aranguren 2701, C1406FWY Buenos Aires, Capital Federal, Argentina	Hospital - Surgery, Emergency, Medicine, Pediatrician, OB/GYN Departments	Public	Urban	336		http://www.hospitalalvarez.org/index.php
	Diagonal 75, La Plata, Buenos Aires Province, Argentina	Children's Hospital	Public	Appears Suburban	350	21	http://www.ludovica.org.ar/inicio.html
	Buenos Aires, Argentina	Mental Health Hospital	?	Urban	?		http://www.buenosaires.gob.ar/hospitaltobargarcia

Hospital Zonal Gral Agudos Dr Isidoro Iriate	Quilmes, Buenos Aires Province, Argentina	Hospital	Public	Urban	221		None but I found this: http://www.argentino.com.ar/hospital-zonal-general-agudos-dr-isidoro-iriarte-F1506C80F18D2
Casa Hospital San Juan de Dios - Ramos Mejia	Ramos Mejía, Buenos Aires Province, Argentina	Hospital	Private	Urban	**78** (http://www.hsjd.org/arg-ramos/centros-arg-ramos.php)		http://www.hsjd.org/hospital/index.php
Hospital Area Programa Choele Choel	Choele Choel, Río Negro Province, Argentina	Hospital	?	?	**70** (http://www.argentino.com.ar/hospital-area-programa-choele-choel-F140FC80713D4)		no website
	Calle 9, 1900 La Plata, Buenos Aires Province, Argentina						
Hospital Espanol de Mendoza	Avenida San Martín 965, Godoy Cruz, Mendoza Province, Argentina	Hospital	Private	Urban	**200** (http://www.hespanol.com.ar/paginas/index/infraestructura)	37	http://www.hespanol.com.ar/paginas/index/mision-vision-y-objetivos
Hospital General de Agudos Carlos Durand	Avenida Díaz Vélez 5044, C1405DCS Buenos Aires, Argentina	Hospital	Public	Urban	**326** (2001 http://www.wilsoncenter.org/sites/default/files/INFORME_HOSPITALES_DE_AGUDO.PDF)		
	San Juan 2021, C1232AAC Buenos Aires, Capital Federal, Argentina						
Hospital General de Agudos Dr Cosme Argerich	Pi y Margall 750, C1155AHD Buenos Aires, Argentina	Hospital	Public	Urban	**400** (http://www.hospitalargerich.org.ar/)		http://www.hospitalargerich.org.ar/
Hospital Privado Centro Medico de Cordoba	Naciones Unidas 346, Córdoba, Córdoba Province, Argentina	Hospital	? (I would guess private)	Urban	**268** (http://www.hospitalprivadosa.com.ar/hospital/infraestructura.htm)	12	
	Río Grande, Argentina						
Hospital Italiano de Cordoba	Roma 550, Córdoba, Córdoba Province, Argentina	Hospital	Private	Urban	**240** (http://www.hospital-italiano.com.ar/hospital-italiano/institucional/info)	31	http://www.hospital-italiano.com.ar/hospital-italiano/

Hospital General de Agudos Enrique Tornu	Combatientes de Malvinas 3002, C1427ARN Buenos Aires, Capital Federal, Argentina	Hospital	Public	Urban	**185** (2001: http://www.wilsoncenter.org/sites/default/files/INFORME_HOSPITALES_DE_AGUDO.PDF)		http://www.buenosaires.gob.ar/areas/salud/sistemas_salud/ficha.php?id=26
	Rosario, Santa Fe Province, Argentina	Pediatric Hospital	Public	Urban	**150** (http://www.fundacionvilela.org/hospital/index.html)		http://www.fundacionvilela.org/hospital/index.html
Hospital Zonal Dr. Ramon Carrillo or Hospital Bariloche	San Carlos de Bariloche, Río Negro Province, Argentina	Hospital - General Medicine, General Surgery, Tocogynecology, Clinical Medicine, Pediatrics and Emergency	Public	Urban	**146** (http://www.hospitalbariloche.com.ar/datos.htm)		http://www.hospitalbariloche.com.ar/
Hospital Central de Mendoza	Gran Hotel Mendoza, Av España 1210, 5500 Mendoza, Mendoza Province, Argentina	Hospital	Public?	Urban/Suburban	**310** (2008: http://www.cepal.org/publicaciones/xml/2/34262/DocW30fin.pdf)		http://www.hospitalcentral.mendoza.gov.ar/
	Italia 1750, Florida, Buenos Aires Province, Argentina	Hospital: Specializes in Respiratory Medicine including Pulmonary Obstructions, Pneumonia, Bronchitis, Lung Trasplants,etc	Public	Suburban	?		http://www.ms.gba.gov.ar/sitios/hcetrangolo/
Hospital Udaondo	Avenida Caseros 2061, Buenos Aires, Buenos Aires Province, Argentina	Gastroenterology Hospital	Public	Urban	**7** (http://hospitaludaondo.org/area%20medica/terapia.html)		http://hospitaludaondo.org/acerca.html
El Hospital Regional Ushuaia - Tierra Del Fuego	Av. 12 de Octubre y Maipú, V9410 Ushuaia, Tierra del Fuego, Argentina	Hospital	Public?	Urban	?		http://www.tierradelfuego.org.ar/funcardio/hospital.htm#arriba
Hospital de Pediatria SAMIC or Hospital Garrahan	Comb D L Pozos 1881, Parque Patricios, Combate de Los Pozos 1881, C 1245 AAM Buenos Aires, Capital Federal, Argentina	Pediatric Hospital	Public	Urban	**644 between the two locations** (http://www.garrahan.gov.ar/indicadores.php)	6	http://www.garrahan.gov.ar/
	2231, B1754AZK, Buenos Aires Province, Argentina	Hospital	Private	Urban	**123** (http://www.hospitalitaliano.org.ar/sanjusto/index.php?contenido=ver_curso.php&id_curso=13370)		http://www.hospitalitaliano.org.ar/sanjusto/

	Salvador Arias, Mendoza, Mendoza Province, Argentina						???? https://plus.google.com/115401639516567393536/about?gl=us&hl=en
	Avenida 51 1725, 1900 La Plata, Buenos Aires Province, Argentina	Hospital	Public?	Urban	?		http://www.italianolaplata.org.ar/
Hospital General de Agudos Dalmacio Velez Sarsfield	Pedro Calderón de la Barça 1550, C1407KQF Buenos Aires, Capital Federal, Argentina	Hospital	Public	Urban	**120** (http://www.wilsoncenter.org/sites/default/files/INFORME_HOSPITALES_DE_AGUDO.PDF)		http://www.buenosaires.gob.ar/areas/salud/sistemas_salud/ficha.php?id=24
Complejo Medico Policial "Churruca-Visca"	Uspallata 3400, 1437 Buenos Aires, Argentina						no website = hard to find any information or whether or not it is actually a functioning hospital
	Avenida Gral. Paz, Lomas del Mirador, Buenos Aires Province, Argentina	Veternary Hospital					
	Cramer 4601, Buenos Aires, Capital Federal, Argentina	Hospital	Private	Urban	**63** (http://www.argentino.com.ar/hospital-privado-de-ninos-fundacion-hospitalaria-F140FC20218D5)		http://www.hpn.org.ar/preguntas-frecuentes.php
	Presidente Perón 450, 3100 Paraná, Entre Ríos Province, Argentina	Hematological Hospital (not clear if it takes patients or is a lab?)				46	http://www.hospitalsanmartin.org.ar/
Hospital de Odontologia "Jose Duenas"	Muñiz 15, Buenos Aires, Buenos Aires Province, Argentina	Orthodontist	Public	Urban	------		http://www.buenosaires.gob.ar/areas/salud/sistemas_salud/ficha.php?id=17
	2401, B8001DDU Bahía Blanca, Buenos Aires Province, Argentina	Hospital	Public	Urban	**360** (http://www.hospitalpenna.com.ar/archivos/hospital_datos.html)		http://www.hospitalpenna.com.ar/
	Hipólito Yrigoyen 1702, Ciudadela, Buenos Aires Province, Argentina						No website, and although I found the google maps page for it, most of the links were for the Hospital General de Agudos de Ramon Carrillo
Hospital de Quemados Dr. Arturo Umberto Illia	Avenida Pedro Goyena 369, C1424BSD Buenos Aires, Argentina	Burn Center	Public	Urban	?		http://www.buenosaires.gob.ar/areas/salud/sistemas_salud/ficha.php?id=19

	Avenida Centenario, Corrientes, Corrientes Province, Argentina						
	Mendoza, Mendoza Province, Argentina	Hospital	Public	Rural	**379** (2008: http://www.cepal.org/publicaciones/xml/2/34262/DocW30fin.pdf)		http://www.hospitallagomaggiore.com/
	Martínez, Buenos Aires Province, Argentina						
	Godoy Cruz 475, Maipú, Mendoza Province, Argentina	Hospital	Public	Suburban/ Rural	**76** (2008: http://www.cepal.org/publicaciones/xml/2/34262/DocW30fin.pdf)		http://www.hospitalparoissien.com.ar.ybsitecenter.com/privacidad
Hospital General de Agudos P Pinero	Varela 1301, C1406ELA Buenos Aires, Capital Federal, Argentina	Hospital	Public	Urban	**326** (http://www.argentino.com.ar/hospital-general-de-agudos-parmenio-t-pinero-F140FC20219D1) or **400** (https://prezi.com/_yukhx3etz-r/hospital-general-de-agudos-dr-parmenio-pineiro/)		http://www.buenosaires.gob.ar/areas/salud/sistemas_salud/ficha.php?id=15
Hospital Zonal Gral de Agudos de Magdalena Villegas de Martinez	Avenida de Los Constituyentes 395, General Pacheco, Buenos Aires Province, Argentina	Hospital	Public	Urban	?		http://www.ms.gba.gov.ar/sitios/hmartinez/
	Parque Chas, Buenos Aires, Buenos Aires Province, Argentina						
	Mainini 240, Florencio Varela, Province de Buenos Aires	Hospital	Public	Suburban/ Rural	?		http://www.hospitalmipueblo.org/
	Mendoza 2152, Villa María, Córdoba Province, Argentina	Hospital	Public	Rural	**114** (http://www.cadena3.com/contenido/2014/12/16/138758.asp)		no website (looks like a relatively new hospital)
Sanatorio Plaza Huincul	Plaza Huincul, Neuquén Province, Argentina	Hospital	Private	Rural	?		No website
Universidad de Buenos Aires, Facultad de Medicina	Paraguay 2250, Buenos Aires, Capital Federal, Argentina	University Medicine Department					
	Calle 2 432, La Plata, Buenos Aires Province, Argentina	Hospital	Private	Urban	**76** (call them seats not beds so not sure: http://www.hospitalsudamericano.com/infraes		http://www.hospitalsudamericano.com/

					tructura.html)		
	Lavalle 383, San Antonio de Areco, Buenos Aires Province, Argentina	City Hall					
	5401, 1888, Buenos Aires Province, Argentina	Hospital	University Public	Suburban	**128** (2014: http://rankings.americaeconomia.com/mejores-clinicas-hospitales-2014/ranking/)	8	http://www.hospitalelcruce.org/home/index.php
	Gral. Roca 1811, Florida, Buenos Aires Province, Argentina	Hospital	Private	Urban	?		http://www.grupog8salud.com.ar/miembros_privadomodelo.html and http://www.hospitalprivadomodelo.com/#!/page_About
Hospital Privado Regional Bariloche	20 de Febrero 598, 8400 San Carlos de Bariloche, Río Negro Province, Argentina	Hospital	Private	Urban/Suburban	**69** (http://www.hprbariloche.com/servicios)		http://www.hprbariloche.com/
	Avenida Rivadavia 22054, 1714 Ituzaingó, Buenos Aires Province, Argentina	Veternary Hospital					
	Pichincha 1890, Buenos Aires, Argentina	Pediatric Hospital	Public	Urban	**644 between the two locations** (http://www.garrahan.gov.ar/indicadores.php)	6	http://www.garrahan.gov.ar/
	Dr. Enrique Finochietto 849, 1272 Ciudad De Buenos Aires, Argentina						
Hospital Regional Espanol Bahia Blanca	Estomba 571, Bahía Blanca, Buenos Aires Province, Argentina	Hospital	?	?	?		no website
El Hospital Municipal Eva Peron (Merlo)	Colón 451, Merlo, Buenos Aires Province, Argentina	Hospital	Public	Urban	**134** (http://www.infoban.com.ar/despachos.asp?cod_des=41692&ID_Seccion=10)		http://www.merlo.gob.ar/detalle.php?t=13&d=3192&a=hospital-municipal-eva-peron
	Sarmiento 3150, Rosario, Santa Fe Province, Argentina	Hospital	Private	Urban	?		no website
	136 2905, Buenos Aires Province, Argentina	Hospital	Public	Urban (on the outskirts of Buenos Aires metropolitan area)	**172** (http://www.argentino.com.ar/hospital-zonal-general-agudos-descentraliz-evita-pueblo-		http://www.evitapueblo.com/

					F1506C70E 1CD0)		
	Necochea 675, Bahía Blanca, Buenos Aires Province, Argentina	Hospital	Private	Urban	?		https://www.hospitalitalianobb.com.ar/
	Avenida Bandera de Los Andes 2603, Guaymallén, Mendoza Province, Argentina	Pediatric Hospital	Public	Suburn/Rural	**238** (2008: http://www.cepal.org/publicaciones/xml/2/34262/DocW30fin.pdf)		No website
	Córdoba, Córdoba Province, Argentina	Hospital	Public	Urban	**38** (http://www.hospitalsanroquecor.com.ar/mm/index.php?option=com_content&view=article&id=63:clinicamedica&catid=69:clinicamedica&Itemid=103)		http://www.hospitalsanroquecor.com.ar/mm/index.php?option=com_content&view=article&id=44:bienvenidos-&catid=1:latest-news&Itemid=50
	Luis Agote 2825, Córdoba, Córdoba Province, Argentina	Hospital	Police Force Personnel	Urban	?		http://www.policiacordoba.gov.ar/
	Fitz Roy, Ushuaia, Tierra del Fuego Province, Argentina						
Hospital de Rehabilitacion Respiratoria Maria Ferrer	Dr. Enrique Finochietto 849, C1272AAA Buenos Aires, Capital Federal, Argentina	Rehabilitation Clinic especially respiration	Public	Urban	**39** (http://www.argentino.com.ar/hospital-de-rehabilitacion-respiratoria-maria-ferrer-F140FC2021BD7)		http://www.buenosaires.gob.ar/areas/salud/sistemas_salud/ficha.php?id=8
Hospital Municipal Dr Hector M Cura	Sarmiento 2669, 7400 Olavarría, Buenos Aires, Argentina	Hospital	Public	Urban	**154** (http://slideplayer.es/slide/1078866/)		http://www.olavarria.gov.ar/
	Boulogne, Buenos Aires Province, Argentina	University Hospital	Public	Urban	**10** (http://www.agencianova.com/nota.asp?n=2004_9_19&id=17222&id_tiponota=11)		http://sanisidro.gob.ar/secretaria-de-salud-publica/hospitales-municipales/hospital-ciudad-de-boulogne/#.VKc9E5NAQe0

Hospital Oftalmologico Dr Pedro Lagleyze	Avenida Juan B. Justo 4151, Buenos Aires, Buenos Aires Province, Argentina	Optomologist	Public	Urban	**36** (http://www.argenpress.info/2010/03/buenos-aires-oculta-cerraron-el.html)		http://www.buenosaires.gob.ar/areas/salud/sistemas_salud/ficha.php?id=27
Hospital General de Agudos Abel Zubizarreta	3952, Buenos Aires, Buenos Aires Province, Argentina	Hosptial	Public	Urban	**99** (2001: http://www.wilsoncenter.org/sites/default/files/INFORME_HOSPITALES_DE_AGUDO.PDF)		http://www.buenosaires.gob.ar/areas/salud/sistemas_salud/ficha.php?id=23
	Góngora 3050, 5000 Córdoba, Córdoba Province, Argentina						No Website
	Coronel Charlone, Buenos Aires Province, Argentina						Is this Hospital Aleman?
	Catamarca 441, Córdoba, Córdoba Province, Argentina	Emergency Medicine Hospital focused on Trauma and High Risk Patients	Public	Urban	**79** (http://www2.cordoba.gov.ar/portal/index.php/secretaria-de-salud/subsec-de-atencion-hospitalaria/hospitales-municipales/hospital-de-urgencias/)		http://www2.cordoba.gov.ar/portal/index.php/secretaria-de-salud/subsec-de-atencion-hospitalaria/hospitales-municipales/hospital-de-urgencias/
Hospital General de Agudos Jose M. Penna	Dr. Prof. Pedro Chutro 3380, C1437JLR Buenos Aires, Argentina	Hospital	Public	Urban	**251** (http://www.argentino.com.ar/hospital-general-de-agudos-dr-jose-m-penna-F140FC2021AD4)		
	Gdor. Ugarte 2152, Olivos, Buenos Aires Province, Argentina	Veternary Hospital					
	1757 Pres. Hipólito Yrigoyen, Vicente López, Buenos Aires Province, Argentina	University but no website so not sure					
Hospital Interzonal Gral. De Agudos San Jose	Bv. Liniers 950, Pergamino, Buenos Aires Province, Argentina	Hosptial	Public	Urban	**206** (http://www.ms.gba.gov.ar/sitios/hsanjose/historia/)		http://www.ms.gba.gov.ar/sitios/hsanjose/
	Paraguay, Buenos Aires, Buenos Aires Province, Argentina					22	Is this the same as row 9? The address is different?

	Ruta Provincial 24, General Rodríguez, Buenos Aires Province, Argentina	Hospital	Public	Urban	?	Urban	http://www.sommer.gov.ar/index.html
	Avenida Díaz Vélez 5044, Buenos Aires, Capital Federal, Argentina	Hospital	Public	Suburban/ Rural	**326** (http://www.wilsoncenter.org/sites/default/files/INFORME_HOSPITALES_DE_AGUDO.PDF)		http://www.hospitaldurand.org.ar/index.html
	Brig. Gral. Juan Manuel de Rosas 5975, Isidro Casanova, Buenos Aires Province, Argentina	Pharmaceuticals Company?(https://www.businessvibes.com/companyprofile/Hospital-dr-Diego-Pariocien)					
	Avenida Don Pedro de Mendoza 1795, C1169AAB Buenos Aires, Capital Federal, Argentina	Dentist Office - specializes in pediatric dentistry	Public	Urban			http://www.buenosaires.gob.ar/areas/salud/sistemas_salud/ficha.php?id=3
Hospital Regional Comodoro Rivadavia	Comodoro Rivadavia, Chubut Province, Argentina	Hospital	Public				No website
	Chubut 368, San Antonio de Padua, Buenos Aires Province, Argentina	Maternity and Child Hospital					No website
Hospital General de Agudos Dr Teodoro Alvarez	Dr. Juan Felipe Aranguren 2701, Buenos Aires, Capital Federal, Argentina	Hospital - Surgery, Emergency, Medicine, Pediatrician, OB/GYN Departments	Public	Urban	336		http://www.hospitalalvarez.org/index.php
	Hungría Hungría 750, San Miguel de Tucumán, Tucumán Province, Argentina	Pediatric Hospital	Public	Urban	**200** (http://www.argentino.com.ar/hospital-del-nino-jesus-F140EC20413D8)		No website
Hospital Dr. F Glasman	Patricios 347, Bahía Blanca, Buenos Aires Province, Argentina	Hospital	Private	Urban	?		http://www.ambb.com.ar/index.php?SESID=d0f3246d19a040e87c79ad7811a8decb&omf_id=0&omf_op=40
	Buenos Aires, Buenos Aires Province, Argentina						No website
	Avenida Luro 6561, Laferrere, Buenos Aires Province, Argentina	Maternity and Child Hospital	Public	Rural	**71** (http://www.argentino.com.ar/hospital-materno-infantil-dra-teresa-luisa-germani-F1506C9071DD1)		No website

Hospital Escuela de Agudos Dr. Ramon Madariaga	Posadas, Misiones Province, Argentina	Hospital	Public	Suburban	?		No website
Hospital Interzonal de Ezeiza	Leandro N.Alem 350, La Union, Ezeiza, Buenos Aires, Argentina, 1803, Buenos Aires Province, Argentina	Hospital	Public	Rural	**180** (http://www.hospitaleurnekian.com.ar/)		http://www.hospitaleurnekian.com.ar/
Hospital de Rehabilitacion M Rocca	Avenida Segurola 1949, C1407AOM Buenos Aires, Argentina	Rehabilitation Hospital	Public	Urban	**80** (http://www.medmun.org.ar/index.php?option=com_content&view=article&id=579:gremiales-prueba&catid=81:mundo-hospitalario-ano-xviii-no155-agosto-2009)		http://www.buenosaires.gob.ar/areas/salud/sistemas_salud/ficha.php?id=25
	Avenida Naciones Unidas 346, Córdoba, Córdoba Province, Argentina	Hospital	? (I would guess private)	Urban	**268** (http://www.hospitalprivadosa.com.ar/hospital/infraestructura.htm)		http://www.hospitalprivadosa.com.ar/
	Gral. Lavalle 2066, José C. Paz, Buenos Aires Province, Argentina	Hospital	Private	Urban	**106** (1998: http://www.hospitalduhau.com.ar/quienes.php)		http://www.hospitalduhau.com.ar/index.php
	Buenos Aires, Argentina	Military Air Force Hospital	Military personnel and their families	Urban	**131** (http://www.hac.mil.ar/website/index.php?option=com_content&view=article&id=95&Itemid=221)		http://www.hac.mil.ar/website/
	Resistencia, Chaco Province, Argentina	Hospital	Public	Suburban	?		http://www.chaco.gov.ar/ministeriosalud/hperrando/index.htm
	Lamadrid, San Miguel de Tucumán, Tucumán Province, Argentina	Hospital					No website
	Ejército Nacional 613, Granada, Miguel Hidalgo, 11520 Mexico City, Federal District, Mexico						
	Juan Badiano 1, Belisario Domínguez, 14080 Tlalpan, Federal District, Mexico						
	Santos Degollado 75, Centro, 45400 Tonalá, Jalisco, Mexico						
	Tlacotalpan 59, Roma Sur, Cuauhtémoc, 06760 Mexico City, Federal District, Mexico						
	Eje 2A Sur (Dr. Balmis) 148, Doctores, Cuauhtémoc, 06726						

	Mexico City, Federal District, Mexico						
	Tarascos 3435, Monraz, 44670 Guadalajara, Jalisco, Mexico						
	Hidalgo 329, Centro, 37000 León, Guanajuato, Mexico						
	Isidro Fabela S/N, María Isabel, 56615 Valle De Chalco Solidaridad, State of Mexico, Mexico						
	Coronel Calderón 777, El Retiro, 44280 Guadalajara, Jalisco, Mexico						
	Avenida Alcalde 2170, Santa Monica, 44220 Guadalajara, Jalisco, Mexico						
	Avenida Paseo de los Héroes 10999, Zona Río, 22010 Tijuana, Baja California, Mexico						
	Felix Cuevas 540, Del Valle, Benito Juarez, 03229 Mexico City, Federal District, Mexico						
	Avenida Insurgentes Sur 3877, La Fama, Tlalpan, 14269 Mexico City, Federal District, Mexico						
	Enrique Díaz de León 238, Americana, 44160 Guadalajara, Jalisco, Mexico						
	Doctor Márquez 162, Doctores, Cuauhtémoc, 06720 Mexico City, Federal District, Mexico						
	Tuxpan 25, Roma Sur, Cuauhtémoc, 06760 Mexico City, Federal District, Mexico						
	Cecilio Robledo 120, Venustiano Carranza, 15960 Federal District, Mexico						
	Blvd Avila Camacho S/N, Lomas de Sotelo, Miguel Hidalgo, 11200 Mexico City, Federal District, Mexico						
	Avenida Universidad 1321, Florida, Álvaro Obregón, 01030 Mexico City, Federal District, Mexico						
	Av Zoquipan 1050, Seattle, 45170 Zapopan, Jalisco, Mexico						
	Av. I. Morones Prieto 3000 PTE., Col. Los Doctores 64710, Monterrey, N.L. México, Mexico						
	Jose Alvarez del Castillo Z. 1542, Country Club, Guadalajara, Jalisco, Mexico						
	Saturnino Herrán 59, San José Insurgentes, Benito Juarez, 03900 Mexico City, Federal District, Mexico						
	Carretera Nacional 6501, La Estanzuela, 64988 Monterrey, Nuevo León, Mexico						

	Ejercito Nacional 613, Granada, 11520 Mexico City, Federal District, Mexico						
	Paseo del Tecnológico 909, Residencial Tecnológico, 27250 Torreon, Coahuila, Mexico						
	Revolución 1182, San José Insurgentes, Benito Juarez, 01000 Mexico City, Federal District, Mexico						
	San Felipe 1014, Sector Hidalgo, 44290 Guadalajara, Jalisco, Mexico						
	Avenida Instituto Politécnico Nacional 5160, Gustavo A. Madero, 07760 Federal District, Mexico						
	Belisario Domínguez 2439, Centro, 64060 Monterrey, Nuevo León, Mexico						
	Vasco de Quiroga 15, Sección XVI, 14000 Mexico City, Federal District, Mexico						
	Árbol del Fuego 80, El Rosario Coyoacán, Coyoacán, 04380 Mexico City, Federal District, Mexico						
	Estaño 307, Felipe Angeles, Venustiano Carranza, 15310 Mexico City, Federal District, Mexico						
	11 Oriente 1826, 72501 Puebla, Mexico						
	Calzada de Tlalpan 4800, Sección XVI, Tlalpan, 14080 Mexico City, Federal District, Mexico						
	Universidad 101, Villas de La Universidad, 20020 Aguascalientes Municipality, Aguascalientes, Mexico						
	Sur 136, 01120 Mexico City, Federal District, Mexico						
	Periférico Sur 5246, Pedregal de Carrasco, Coyoacán, 04700 Mexico City, Federal District, Mexico						
	Ecuador 2331, Balcones de Galerías, 64620 Monterrey, Nuevo León, Mexico						
	Avenida Gabriel Mancera 222, Del Valle, Benito Juarez, 03020 Ciudad de México, Federal District, Mexico						
	Ezequiel Montes 135, Tabacalera, Cuauhtémoc, 06030 Mexico City, Federal District, Mexico						
	Avenida Chapultepec 489, Juárez, Cuauhtémoc, 06600 Ciudad, Federal District, Mexico						
	Boulevard El Niño Poblano 5307, Concepción La Cruz, 72190 Puebla, Mexico						

	Del Hueso S/N, Ex-Hacienda Coapa, Coyoacan, 14310 Ciudad de Mexico, Federal District, Mexico						
	Nicolás Copérnico 4000, Las Arboledas, 44550 Zapopan, Jalisco, Mexico						
	Nueva York, Nápoles, Benito Juarez, 03810 Mexico City, Federal District, Mexico						
	Independencia Oriente, Guadalajara, Jalisco, Mexico						
	Vito Alessio Robles 23, Guadalupe Chimalistac, Álvaro Obregón, 01030 Mexico City, Federal District, Mexico						
	Trabajadores de Agricultura s/n, Fovissste Morelos, 58120 Morelia, Michoacán, Mexico						
	Tlahuac 4866, San Lorenzo Tezonco, Iztapalapa, 09790 Mexico City, Federal District, Mexico						
	Agrarismo 208, Escandón, Miguel Hidalgo, 11800 Mexico City, Federal District, Mexico						
	20 de Noviembre 82, Centro, Cuauhtémoc, 06090 Mexico City, Federal District, Mexico						
	Durango 296, Roma, Cuauhtémoc, 06700 Mexico City, Federal District, Mexico						
	Insurgentes Sur 3700 Letra C, Insurgentes Cuicuilco, Coyoacán, 04530 Mexico City, Federal District, Mexico						
	Pablo Valdez 719, San Juan de Dios, 44360 Guadalajara, Jalisco, Mexico						
	Circunvalación 6, Jardines de Querétaro, 76020 Santiago de Querétaro, Querétaro, Mexico						
	Calle Camino Santa Teresa 1055, Heroes de Padierna, Magdalena Contreras, 10700 Mexico City, Federal District, Mexico						
	Pedro Moreno 934, 44140 Guadalajara, Jalisco, Mexico						
	Avenida Hidalgo 1900, Altavista, 89240 Tampico, Tamaulipas, Mexico						
	Centro, 68000 Oaxaca, Mexico						
	Terranova 556, Colonia Providencia, 44647 Guadalajara, Jalisco, Mexico						
	Prolongación Reforma Carlos Graff Fernández 154, Tlaxcala, Cuajimalpa, 05300 Mexico City, Federal District, Mexico						
	Doctor Ignacio Morones Prieto 3000, Sertoma, 64710 Monterrey, Nuevo León,						

	Mexico						
	Camino al Estadio Azteca 179, El Caracol, Coyoacán, 04739 Tlalpan, Federal District, Mexico						
	Ave. México 2, Tizapan, Álvaro Obregón, 01080 Federal District, Mexico						
	Boulevard Lázaro Cárdenas SN, El Medano Ejidal, 23410 Cabo San Lucas, Baja California Sur, Mexico						
	Presa El Tintero 764, San Joaquín, 44770 Guadalajara, Jalisco, Mexico						
	Constituyentes 1075, Moderna, 44190 Guadalajara, Jalisco, Mexico						
	Justo Sierra 2130, Ladrón de Guevara, 44600 Guadalajara, Jalisco, Mexico						
	Francisco I Madero Sur 59, Centro, 27000 Torreon, Coahuila, Mexico						
	Calzada Legaria 371, México Nuevo, Miguel Hidalgo, 11260 Mexico City, Federal District, Mexico						
	Tolnahuac 14, San Simon Tolnahuac, Cuauhtémoc, 06920 Mexico City, Federal District, Mexico						
	Calzada Mexico-Xochimilco 289, 14389 Tlalpan, Federal District, Mexico						
	Prado Sur 800, Lomas de Chapultepec, Miguel Hidalgo, 11000 Mexico City, Federal District, Mexico						
	Av. Carlos Graef Fernandez 154, Tlaxala, Cuajimalpa de Morelos, 05300 Mexico City, Federal District, Mexico						
	Tiburcio Garza Zamora Kilómetro 5.5, Rancho Grande, 88610 Reynosa, Tamaulipas, Mexico						
	Guadalajara, Jalisco, Mexico						
	Sur 136 116, Las Americas, Álvaro Obregón, 01120 Mexico City, Federal District, Mexico						
	20 De Noviembre 1074, 91700 Veracruz, Mexico						
	Pino Suarez 645, Centro, 64000 Monterrey, Nuevo León, Mexico						
	Campo Matillas, San Antonio, Azcapotzalco, Mexico City, Federal District, Mexico						
	Hidalgo 1351, Americana, 44100 Guadalajara, Jalisco, Mexico						

	Av. Cuauhtémoc 330, Doctores, Cuauhtémoc, 06720 Mexico City, Federal District, Mexico						
	Avenida Chapultepec 500, Roma, Cuauhtémoc, 06700 Mexico City, Federal District, Mexico						
	Vialidad de La Barranca 22, Valle de Las Palmas, 52763 Huixquilucan de Degollado, State of Mexico, Mexico						
	Pradera 1101, Azteca, 37520 León, Guanajuato, Mexico						
	Avenida Plutarco Elías Calles 473, Santa Anita, Iztacalco, 08300 Mexico City, Federal District, Mexico						
	Ávenida Tulum Sur 260, Mz 4,5,9, Sm 7, Benito Juárez, 77500 Cancún, Quintana Roo, Mexico						
	Calle 20 de Enero 927, Centro, 37320 León, Guanajuato, Mexico						
	Gregorio V. Gelati 29, San Miguel Chapultepec, Miguel Hidalgo, 11850 Mexico City, Federal District, Mexico						
	Campos Eliseos 9371, Fracc. Campos Eliseos, 32472 Ciudad Juárez, Chihuahua, Mexico						
	Calzada Ignacio Zaragoza 185, Venustiano Carranza, 15900 Federal District, Mexico						
	Aquiles Serdán 151, San Miguel El Alto Centro, 47140 San Miguel el Alto, Jalisco, Mexico						
	López Mateos Norte 766, 44600 Guadalajara, Jalisco, Mexico						
	Joaquin Pardave 32, Jorge Negrete, Gustavo A. Madero, 07280 Mexico City, Federal District, Mexico						
	Santa Teresa 1055, Heroes de Padierna, Magdalena Contreras, 10700 Mexico City, Federal District, Mexico						
	Reforma 1000, 21100 Mexicali, Baja California, Mexico						
	Veracruz, Jardín, Nuevo Laredo, Tamaulipas, Mexico						
	Aboceta 151, Álvaro Obregón, 1140 Federal District, Mexico						
	Xontepec 127, Toriello Guerra, Tlalpan, 14050 Mexico City, Federal District, Mexico	Hospital (CHECK)	Private	Urban (CHECK)	314 (2014: http://rankings.americaeconomia.com/mejores-clinicas-hospitales-2014/rankin		

					g/)		
	Astrónomos, Escandón, Miguel Hidalgo, Mexico City, Federal District, Mexico						
	Vasco Nunez de Balboa 1003, Fracc. Hornos, 39355 Acapulco, Guerrero, Mexico						
	Eje 2A Sur 143, Roma Norte, Cuauhtémoc, 06700 Mexico City, Federal District, Mexico						
	Avenida Instituto Politécnico Nacional esq Eje Fortuna S/n, Magdalena de Las Salinas, Gustavo A. Madero, 07300 Mexico City, Federal District, Mexico						
	Tuxpan 400, Mitras Norte, 64320 Monterrey, Nuevo León, Mexico						
	Boulevard José María Morelos y Pavón 340, Bachoco, 83148 Hermosillo, Sonora, Mexico						
	Parroquia de San Vicente Ferrer, Av 2 64, San Pedro de Los Pinos, Benito Juarez, 03020 Mexico City, Federal District, Mexico						
	Avenida Francisco I. Madero 1060, Nueva, 21100 Mexicali, Baja California, Mexico						
	Francisco Villa, Doctores, Matamoros, Tamaulipas, Mexico						
	República de Perú 102, Fraccionamiento Las Américas, 20293 Aguascalientes, Mexico						
	Calzada Mexico Xochimilco 101, 14370 Tlalpan, Federal District, Mexico						
	Cll De Las Arenas 151-A, 22880 Ensenada Municipality, Baja California, Mexico						
	Donato Guerra 619, 44100 Guadalajara, Jalisco, Mexico						
	Río Bamba 639, Magdalena de Las Salinas, Gustavo A. Madero, 07760 Mexico City, Federal District, Mexico						
	No Reeleccion 101, Centro, 85000 Ciudad Obregón, Sonora, Mexico						
	Ferrocarril Central 709, Bajío de Las Americas, 38020 Celaya, Guanajuato, Mexico						
	Guanajuato-Silao Kilómetro 6.5, Los Alcaldes, 36250 Guanajuato, Mexico						
	Eje 7 Sur (Municipio Libre) 270, Portales, Benito Juarez, 03300 Mexico City, Federal						

	District, Mexico						
	Vicente García Torres 46, Barrio San Lucas, Coyoacán, 04030 Mexico City, Federal District, Mexico						
	2085, El Espejo II, Villahermosa, Tabasco, Mexico						
	Vicuña, Atacama Region, Chile						
	Providencia, Santiago, Chile						
	Huérfanos 3255, Santiago, Chile						
	Santos Dumont 999, Santiago, Chile						
	Ramón Barros Luco 3301, Santiago Metropolitan Region, Chile						
	Tomé, Biobío Region, Chile						
	Santiago, Chile						
	San Antonio, Valparaíso Region, Chile						
	Avenida Los Torres 5150, Santiago Metropolitan Region, Chile						
	Calle Alvarez 662, Valparaíso, Valparaíso Region, Chile						
	Av Holanda 50, Santiago Metropolitan Region, Chile						
	Vitacura 5951, Vitacura, Santiago Metropolitan Region, Chile	Hospital (CHECK)	Private	Urban	426 (2014: http://rankings.americaeconomia.com/mejores-clinicas-hospitales-2014/ranking/)		
	Calle Ilabaca 752, Angol, Araucanía Region, Chile						
	Pedro Aguirre Cerda, Santiago Metropolitan Region, Chile		Public		146 (2014: http://rankings.americaeconomia.com/mejores-clinicas-hospitales-2014/ranking/)		
	Chimbarongo, O'Higgins Region, Chile						
	Avenida Santa Rosa 1234, Santiago Metropolitan Region, Chile						
	Las Condes, Santiago Metropolitan Region, Chile						
	1 Norte, Maule Region, Chile						

	Calle San Jose 301, Puerto Montt, Los Lagos Region, Chile						
	Concha Y Toro, O'Higgins Region, Chile						
	Avenue Roosevelt, Biobío Region, Chile						
	Alvarez Av. Álvarez, 1532, Valparaíso Region, Chile						
	Avda Alejandro Fleming 7885, Las Condes, Santiago, Santiago Metropolitan Region, Chile						
	Profesor Alberto Zañartu 1085, Santiago Metropolitan Region, Chile						
	Calle Vital Apoquindo 1200, P 1, Santiago Metropolitan Region, Chile						
	Marcoleta 367, Santiago Metropolitan Region, Chile						
	Pje 12, Santiago Metropolitan Region, Chile						
	Avenida Concha Y Toro 3459, Puente Alto, Santiago Metropolitan Region, Chile	General Hospital	Public		777 (2014: http://rankings.americaeconomia.com/mejores-clinicas-hospitales-2014/ranking/)		
	Av Antonio Varas 360, Providencia, Santiago Metropolitan Region, Chile						
	Calle Comercio 632, Puente Alto, Santiago Metropolitan Region, Chile						
	Mar Tirreno 3349, Santiago Metropolitan Region, Chile	Hospital (CHECK)	University Public		607 (2011: http://rankings.americaeconomia.com/mejores-clinicas-hospitales-2014/ranking/)		
	Valparaíso, Valparaíso Region, Chile						
	Valenzuela Llanos, Santiago, Chile						
	Las Condes, Chile						
	Gran Avenida José Miguel Carrera 3204, Chile						
	Doctor Moukarzel, Coquimbo Region, Chile						
	San Martín, Biobío Region, Chile						
	Calle Limache 1667, Valparaíso, Valparaíso Region, Chile						
	Calle Miraflores 2085, San Felipe, Valparaíso Region, Chile						

	Avenida Recoleta 464, Santiago Metropolitan Region, Chile						
	Talca, Maule Region, Chile						
	Avda Sta Maria 1810, Providencia, Santiago, Santiago Metropolitan Region, Chile						
	Poblacion Kennedy, Biobío Region, Chile						
	Calle Juan Jimenez, O'Higgins Region, Chile						
	Santa Julia 870, Santiago Metropolitan Region, Chile						
	Titan, Santiago, Chile						
	Av Domingo Santa María 1196, Santiago Metropolitan Region, Chile						
	Valparaíso, Valparaíso Region, Chile						
	Calle San Pablo 6159, 6106, Lo Prado, Santiago, Santiago Metropolitan Region, Chile						
	Calle Bellavista 276, Providencia, Santiago, Santiago Metropolitan Region, Chile						
	Avenida Tabancura, Santiago Metropolitan Region, Chile						
	Limache 1741, Valparaíso Region, Chile						
	Blanco, Araucanía Region, Chile						
	Vista Hermosa, O'Higgins Region, Chile						
	Avenida Camilo Henríquez 2451, Puente Alto, Santiago Metropolitan Region, Chile						
	Manson de Velasco, Maule Region, Chile						
	Calle Serafin Zamora 190, Santiago, Santiago Metropolitan Region, Chile						
	Avda Argentina 1962, Antofagasta Region, Chile						
	Santa María 500, Santiago 7520378, Región Metropolitana, Chile						
	Calle Limache 1741, Valparaíso, Valparaíso Region, Chile						
	Videla, Coquimbo, Coquimbo Region, Chile						
	Calle Subida Alessandri, Valparaíso, Valparaíso Region, Chile						
	Avda Pedro de Valdivia 2219, D 1, Providencia, Santiago, Santiago Metropolitan Region, Chile						
	Avda Diego Silva Henriquez 1690, Santiago, Santiago Metropolitan Region, Chile						

	Calle las Hualtatas 5951, Vitacura, Santiago, Santiago Metropolitan Region, Chile						
	Calle Profesor Alberto Zanartu 1085, Independencia, Santiago, Santiago Metropolitan Region, Chile						
	Avenida Atlántico Nº 4050 - 3º Sector- Gómez Carreño, Viña del Mar 2540934, Quinta Región de Valparaíso, Chile						
	Avda Antonio Varas 245, Providencia, Santiago, Santiago Metropolitan Region, Chile						
	Calle Mac Iver, Galvarino, Araucanía Region, Chile						
	Calle Sierra Bella 1181, 87, Santiago, Santiago Metropolitan Region, Chile						
	Avda Recoleta 2774, Recoleta, Santiago, Santiago Metropolitan Region, Chile						
	Calle Enrique Mac Iver 498, Temuco, Araucanía Region, Chile						
	Valencia, Chile						
	Chile						
	Avda Jose Miguel Claro 988, Providencia, Santiago, Santiago Metropolitan Region, Chile						
	Calle Subida Leopoldo Carvallo 200, Valparaíso, Valparaíso Region, Chile						
	Calle Rancagua 878, Providencia, Santiago, Santiago Metropolitan Region, Chile						
	Avda Salvador 130, D P, Providencia, Santiago, Santiago Metropolitan Region, Chile						
	Avda Alto Horno 777, Concepción, Biobío Region, Chile						
	San Bernardo, Santiago Metropolitan Region, Chile						
	Avda 11 de Septiembre 1945, Of 308, Providencia, Santiago, Santiago Metropolitan Region, Chile						
	Avda los Pajaritos 2470, Maipu, Santiago, Santiago Metropolitan Region, Chile						
	Avda Tomas Moro 710, Santiago, Santiago Metropolitan Region, Chile						
	J Manuel Balmaceda, Coquimbo Region, Chile						
	Estacion Central, Santiago Metropolitan Region, Chile						
	Avda Eliodoro Yanez 2274, Providencia, Santiago, Santiago Metropolitan						

	Region, Chile						
	Calle Eulogio Goycolea 450, S A, Puerto Montt, Los Lagos Region, Chile						
	Iquique, Tarapacá Region, Chile						
	Calle San Martin 1270, Valparaíso, Valparaíso Region, Chile						
	Calle Tristan Valdes 66, Maipu, Santiago, Santiago Metropolitan Region, Chile						
	El Bosque, Santiago Metropolitan Region, Chile						
	Calle Julio Cesar 10905, Santiago Metropolitan Region, Chile						
	Psje Paula Jaraquemada 251, Santiago Metropolitan Region, Chile						
	Los Carrera 299, Concepción, Biobío Region, Chile						
	Calle Carrera 702, Concepción, Biobío Region, Chile						
	Avda Pedro de Valdivia 801, Concepción, Biobío Region, Chile						
	San Martín 711, Santiago Metropolitan Region, Chile						
	Av Las Condes Av. Las Condes 8631, Chile						
	Calle Jose Manuel Balmaceda 4420, Consultorio Renca, Renca, Santiago, Santiago Metropolitan Region, Chile						
	Calle Pdte Prieto 118, Santiago Metropolitan Region, Chile						
	Avda Luis Pasteur 5292, Vitacura, Santiago, Santiago Metropolitan Region, Chile						
	Calle Almte Pastene 71, Providencia, Santiago, Santiago Metropolitan Region, Chile						
	Curacautín, Araucanía Region, Chile						
	Santiago, Las Condes, Santiago Metropolitan Region, Chile						
	Calle Janequeo 5662, Quinta Normal, Santiago, Santiago Metropolitan Region, Chile						
	Avda Liber Bernardo O`Higgins 1620, Santiago, Santiago Metropolitan Region, Chile						
	Uruguay 325 - Casilla 5D Pucón, Pucón 4920246, Novena Región de La Araucanía, Chile						
	Avda Sta Rosa 1448, 91, Santiago, Santiago Metropolitan Region, Chile						

	Avda Sta Maria 410, Cesado, Recoleta, Santiago, Santiago Metropolitan Region, Chile						
	Calle Copiapo 1323, Santiago, Santiago Metropolitan Region, Chile						
	21 de Mayo, Arica and Parinacota Region, Chile						
	Calle Asturias 349, Santiago, Santiago Metropolitan Region, Chile						
	Avda Jose Manuel Infante 553, Providencia, Santiago, Santiago Metropolitan Region, Chile						
	Avda Vicuna Mackenna 152, Providencia, Santiago, Santiago Metropolitan Region, Chile						
	Psje el Pajar 10675, El Bosque, Santiago Metropolitan Region, Chile						
	Calle San Isidro 231, Santiago, Santiago Metropolitan Region, Chile						
	Calle Placer 1410, Santiago, Santiago Metropolitan Region, Chile						
	Calle Padre Orellana 1706, Santiago, Santiago Metropolitan Region, Chile						
	Calle Tte Cruz 1087, Lo Prado, Santiago, Santiago Metropolitan Region, Chile						
	Calle el Aguilucho 3440, Providencia, Santiago, Santiago Metropolitan Region, Chile						
http://hospitals.webometrics.info/en/Latin_America/Argentina							
http://www.wilsoncenter.org/sites/default/files/INFORME_HOSPITALES_DE_AGUDO.PDF							
https://salud.santafe.gov.ar/sims/subportal/nomina/nomina.php							

http://www.cepal.org/publicaciones/xml/2/34262/DocW30fin.pdf							
http://www.msal.gov.ar/images/stories/pdf/listado-estab.fert.asistida-1-10-2014.pdf							

Table 2. List of hospitals used in the analysis described in this study.

Hosp_ID	Country	Hospital_Name	Hospital.Address	Latitude	Longitude
ARG_103	ARG	Hospital Central	Mart'nez, Buenos Aires Province, Argentina	-34.477079	-58.510462
ARG_114	ARG	Hospital El Cruce S.A.M.I.C.	5401, 1888, Buenos Aires Province, Argentina	-34.771983	-58.270551
ARG_124	ARG	Hospital Italiano Regional del Sur	Necochea 675, Bah'a Blanca, Buenos Aires Province, Argentina	-38.732858	-62.233483
ARG_128	ARG	Hospital	Fitz Roy, Ushuaia, Tierra del Fuego Province, Argentina	-54.813899	-68.323733
ARG_131	ARG	Hospital Municipal de Boulogne	Boulogne, Buenos Aires Province, Argentina	-34.501017	-58.558339
ARG_146	ARG	Hospital Regional	Comodoro Rivadavia, Chubut Province, Argentina	-45.868389	-67.487319
ARG_154	ARG	Hospital Ezeiza	Leandro N.Alem 350, La Union, Ezeiza, Buenos Aires, Argentina, 1803, Buenos Aires Province, Argentina	-34.866441	-58.534261
ARG_31	ARG	Hospital San Juan de Dios	Ramos Mej'a, Buenos Aires Province, Argentina	-34.639091	-58.55508
ARG_42	ARG	Hospital Nacional Alejandro Posadas	Avenida Pres. Arturo U. Illia, El Palomar, Buenos Aires Province, Argentina	-34.629235	-58.574881
ARG_49	ARG	Hospital Universitario Austral	Avenida Juan Domingo Per—n 1500, 1629, Buenos Aires Province, Argentina	-34.455983	-58.864489
ARG_79	ARG	Hospital	R'o Grande, Argentina	-53.782056	-67.699445
ARG_84	ARG	Hospital Central	Gran Hotel Mendoza, Av Espa–a 1210, 5500 Mendoza, Mendoza Province, Argentina	-32.891801	-68.833188
ARG_90	ARG	Hospital el Carmen	Salvador Arias, Mendoza, Mendoza Province, Argentina	-32.92709	-68.859479

ARG_93	ARG	Hospital churruca	Buenos Aires, Buenos Aires Province, Argentina	-34.640758	-58.410759
ARG_99	ARG	Hospital Ramon Carrillo	Hip—lito Yrigoyen 1702, Ciudadela, Buenos Aires Province, Argentina	-34.626483	-58.555807
CHI_111	CHI	Hospital	21 de Mayo, Arica and Parinacota Region, Chile	-18.482728	-70.313086
CHI_14	CHI	Hospital Padre Hurtado	Pedro Aguirre Cerda, Santiago Metropolitan Region, Chile	-33.553041	-70.634709
CHI_22	CHI	Hospital Gustavo Fricke	Alvarez Av. çlvarez, 1532, Valpara'so Region, Chile	-33.0293	-71.54177
CHI_23	CHI	ClÁnica Cordillera	Avda Alejandro Fleming 7885, Las Condes, Santiago, Santiago Metropolitan Region, Chile	-33.425415	-70.550483
CHI_28	CHI	Hospital Sotero Del Rio	Avenida Concha Y Toro 3459, Puente Alto, Santiago Metropolitan Region, Chile	-33.577353	-70.580883
CHI_34	CHI	Hospital de Carabineros	Las Condes, Regi—n Metropolitana, Chile	-33.41762	-70.527914
CHI_47	CHI	Hospital San Jos	Av Domingo Santa Mar'a 1196, Santiago Metropolitan Region, Chile	-33.41728	-70.653315
CHI_62	CHI	Hospital Naval Almirante Nef	Calle Subida Alessandri, Valpara'so, Valpara'so Region, Chile	-32.997817	-71.544364
CHI_66	CHI	Corp de Amigos del Hospital Roberto del RA-o	Calle Profesor Alberto Zanartu 1085, Independencia, Santiago, Santiago Metropolitan Region, Chile	-33.417305	-70.653567
CHI_74	CHI	Hospital	Alvarez Av. çlvarez, 1532 Valpara'so Region, Chile	-33.029156	-71.542063
CHI_80	CHI	Hospital San Bernardo	San Bernardo, Santiago Metropolitan Region, Chile	-33.592498	-70.696974
CHI_84	CHI	Capilla del Hospital San Juan de Dios	J Manuel Balmaceda, Coquimbo Region, Chile	-29.908259	-71.249533
CHI_88	CHI	Hospital	Iquique, Tarapac‡ Region, Chile	-20.215046	-70.137975
MEX_100	MEX	Medica Sur	Xontepec 127, Toriello Guerra, Tlalpan, 14050 Mexico City, Federal District, Mexico	19.296904	-99.161565
MEX_106	MEX	Hospital San JosAc	Boulevard JosŽ Mar'a Morelos y Pav—n 340, Bachoco, 83148 Hermosillo, Sonora, Mexico	29.120643	-110.952039
MEX_109	MEX	Hospital San Charbel	Francisco Villa, Doctores, Matamoros, Tamaulipas, Mexico	25.869194	-97.481038
MEX_114	MEX	Angeles Lindavista	R'o Bamba 639, Magdalena de Las Salinas, Gustavo A. Madero, 07760 Mexico City, Federal District, Mexico	19.486856	-99.129581
MEX_116	MEX	Medica Avanzada Celaya - Medica Avanzada Contigo	Ferrocarril Central 709, Baj'o de Las Americas, 38020 Celaya, Guanajuato, Mexico	20.529524	-100.829886
MEX_117	MEX	Hospital General de Guanajuato	Guanajuato-Silao Kil—metro 6.5, Los Alcaldes, 36250 Guanajuato, Mexico	20.980877	-101.289292

MEX_120	MEX	Hospital Angeles Villahermosa	2085, El Espejo II, Villahermosa, Tabasco, Mexico	17.996494	-92.953185
MEX_21	MEX	Hospital San Jos	Av. I. Morones Prieto 3000 PTE., Col. Los Doctores 64710, Monterrey, N.L. MŽxico, Mexico	25.668615	-100.35181
MEX_24	MEX	Hospital Christus Muguerza	Carretera Nacional 6501, La Estanzuela, 64988 Monterrey, Nuevo Le—n, Mexico	25.589009	-100.258077
MEX_25	MEX	Hospital EspaA–ol Sociedad de Beneficencia EspaA–ola I.a.p. ComitAc de Damas	Ejercito Nacional 613, Granada, 11520 Mexico City, Federal District, Mexico	19.438509	-99.195106
MEX_26	MEX	Hospital ngeles	Paseo del Tecnol—gico 909, Residencial Tecnol—gico, 27250 Torreon, Coahuila, Mexico	25.517152	-103.395931
MEX_30	MEX	Hospital Sierra Madre	Belisario Dom’nguez 2439, Centro, 64060 Monterrey, Nuevo Le—n, Mexico	25.673395	-100.347862
MEX_32	MEX	Hospital HMG Coyoacn	çrbol del Fuego 80, El Rosario Coyoac‡n, Coyoac‡n, 04380 Mexico City, Federal District, Mexico	19.327234	-99.144877
MEX_36	MEX	Star Medica	Universidad 101, Villas de La Universidad, 20020 Aguascalientes Municipality, Aguascalientes, Mexico	21.897813	-102.304208
MEX_38	MEX	Sedna Hospital	PerifŽrico Sur 5246, Pedregal de Carrasco, Coyoac‡n, 04700 Mexico City, Federal District, Mexico	19.303305	-99.174559
MEX_45	MEX	Hospital Arboledas	Nicol‡s CopŽrnico 4000, Las Arboledas, 44550 Zapopan, Jalisco, Mexico	20.630464	-103.423654
MEX_50	MEX	Hospital Belizario DomÁnguez	Tlahuac 4866, San Lorenzo Tezonco, Iztapalapa, 09790 Mexico City, Federal District, Mexico	19.306797	-99.065081
MEX_57	MEX	Hospital Angeles	Calle Camino Santa Teresa 1055, Heroes de Padierna, Magdalena Contreras, 10700 Mexico City, Federal District, Mexico	19.31149	-99.22135
MEX_63	MEX	Hospital San Jose Tec de Monterrey	Doctor Ignacio Morones Prieto 3000, Sertoma, 64710 Monterrey, Nuevo Le—n, Mexico	25.669652	-100.35175
MEX_66	MEX	Hospital Amerimed Cabo San Lucas	Boulevard L‡zaro C‡rdenas SN, El Medano Ejidal, 23410 Cabo San Lucas, Baja California Sur, Mexico	22.891323	-109.908231
MEX_76	MEX	Christus Muguerza Hospital Reynosa	Tiburcio Garza Zamora Kil—metro 5.5, Rancho Grande, 88610 Reynosa, Tamaulipas, Mexico	26.110316	-98.329832
MEX_81	MEX	Hospital Central	Campo Matillas, San Antonio,	19.479858	-99.200504

		Norte de Pemex	Azcapotzalco, Mexico City, Federal District, Mexico		
MEX_85	MEX	Hospital Angeles Lomas	Vialidad de La Barranca 22, Valle de Las Palmas, 52763 Huixquilucan de Degollado, State of Mexico, Mexico	19.394675	-99.282247
MEX_86	MEX	Issste Hospital Regional Len	Pradera 1101, Azteca, 37520 Le—n, Guanajuato, Mexico	21.09784	-101.6497
MEX_87	MEX	IMSS Hospital General N£mero 30	Avenida Plutarco El'as Calles 473, Santa Anita, Iztacalco, 08300 Mexico City, Federal District, Mexico	19.398736	-99.11758
MEX_91	MEX	Hospital Angeles	Campos Eliseos 9371, Fracc. Campos Eliseos, 32472 Ciudad Ju‡rez, Chihuahua, Mexico	31.714587	-106.393249
